# Controlling Lithium Surface Diffusivity via 2D PtTe_2_, PdTe_2_, and NiTe_2_ Coatings for Anode‐Free and Lithium Metal Batteries

**DOI:** 10.1002/adma.202501261

**Published:** 2025-06-01

**Authors:** Chae Yoon Im, Ga Yeon Lee, Jong Gyeom Kim, Jeong Ho Choi, Suk Jun Kim

**Affiliations:** ^1^ School of Energy Materials and Chemical Engineering Korea University of Technology and Education Cheonan 31253 South Korea

**Keywords:** anode‐free lithium‐ion batteries, lithium metal batteries, surface diffusivities, thin‐film coatings, transition metal dichalcogenides

## Abstract

Anode‐free Li‐ion batteries (AFLBs) and Li‐metal batteries (LMBs) offer superior energy densities compared to conventional Li‐ion batteries with graphite anodes. However, they degrade faster owing to their lower Coulombic efficiency, primarily caused by uneven Li deposition on the current collector (CC) in AFLBs or the Li‐metal anode (LMA) in LMBs. Coating CCs and LMAs has emerged as a promising strategy to enhance the CE. Coating CCs and LMAs with PtTe_2_, PdTe_2_, and NiTe_2_—metallic 2D transition metal dichalcogenides—reveals the critical factors for achieving uniform Li plating. The PtTe_2_ coating facilitates rapid Li surface diffusivity, while the PdTe_2_ and NiTe_2_ coatings provide shorter diffusion paths for Li adatoms on the CCs and LMAs. In addition, Li_2_Te, formed as a byproduct of the decomposition of PdTe_2_ and NiTe_2_ during Li plating, reduces the critical nucleus size by minimizing the interfacial energy between the electrolyte and the plated Li. PtTe_2_ more effectively enhances the AFLB cycling performance, whereas PdTe_2_ and NiTe_2_ are more advantageous for LMBs. Notably, a 5‐nm‐thick PdTe_2_ coating on the LMA achieves 80% capacity retention after 450 cycles using a LiFePO_4_ cathode (3 mAh cm^−2^) at a 0.5 C‐rate.

## Graphical abstract

Lithium plating mechanisms on 2D‐coated current collectors: PtTe_2_ coating enables rapid Li surface diffusivity, while PdTe_2_ and NiTe_2_ coatings shorten diffusion paths for Li adatoms. In addition, Li_2_Te, formed from PdTe_2_ and NiTe_2_ decomposition, reduces the critical nucleus size by lowering the interfacial energy between the electrolyte and deposited Li.

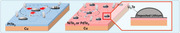

## 1 Introduction

The energy density of rechargeable batteries is crucial for improving the driving range of electric vehicles. Anode‐free Li‐ion batteries (AFLBs) and Li‐metal batteries (LMBs) are considered promising solutions for extending their driving range.^[^ ^1^, ^2^ ^]^ Only a current collector (CC) is used on the anode side in AFLBs without any anodic active materials. Before cycling, Li exists only in the cathode. The complete removal of the anodic active materials dramatically reduces the weight and volume of the cell, and the gravimetric and volumetric energy densities of AFLBs are 1.5‐ and 2‐fold higher, respectively, than those of general Li‐ion batteries (LIBs).^[^ ^3^ ^]^ Lithium metal foil is employed as the Li‐metal anode (LMA) in LMBs to replace graphite, which is the conventional active material used in traditional LIBs. Owing to this substitution, both the volumetric and gravimetric energy densities of LMBs are ≈1.5‐fold higher than those of LIBs. Despite their outstanding energy densities, the low Coulombic efficiencies (CEs) of AFLBs and LMBs have hindered their commercialization. Their low CE is attributed to lithium plating the LMAs and CCs during charging instead of intercalating into the anodic active materials. The non‐uniform electric field on the surface of LMAs and CCs due to the non‐uniform electrical resistance and surface morphology leads to irregular deposition of Li,^[^ ^4^ ^]^ which increases the interfacial area between the electrolyte and deposited Li. A fragile solid‐electrolyte interface (SEI) repeatedly forms at the interface, consuming Li ions and electrolyte with continuous cycling, leading to low CE.^[^ ^5^ ^]^ Various methods have been proposed to improve the CE, including coating the CC with lithiophilic materials, which leads to uniform Li plating.^[^ ^6^, ^7^, ^8^, ^9^ ^]^ Among the various organic and inorganic coating materials, two‐dimensional (2D) TMDs are reportedly promising candidates for coating layers in LMBs and AFLBs.^[^ ^10^, ^11^ ^]^ Two‐dimensional TMD materials have the general formula of MX_2_, where M represents a metal and X is S, Se, or Te. These materials exhibit a layered structure similar to those of graphite and LiCoO_2_, which are commonly used as anodic and cathodic active materials, respectively, in LIBs. Owing to their unique atomic structures, TMDs, such as TiS_2_ and MoS_2_, have been evaluated as cathodic and anodic active materials in LIBs.^[^ ^12^, ^13^ ^]^ In our previous work, we demonstrated that a metallic PdTe_2_ coating on the CC markedly enhances the cycling performance of AFLBs by substantially reducing the nucleation overpotential (*η*_n_) and stabilizing Li plating.^[^ ^10^ ^]^ However, the mechanisms underlying these beneficial effects of PdTe_2_ require further investigation.

The electrochemical performance of AFLBs and LMBs fabricated using PdTe_2_‐, PtTe_2_‐, and NiTe_2_‐coated CCs and LMAs were investigated in this study. A comparative analysis of the cycling performance of the cells and the morphology of the Li deposits on the 2D material coatings during the early stages of charging successfully revealed the key factors driving the enhanced AFLB and LMB performances. 2D‐coated CCs and LMAs, as compared with the bare CCs and LMAs, significantly improved the cycling performance of the cells. Both experimental and numerical analyses revealed that the 2D coatings influence the crystallization overpotential, charge‐transfer resistance, and formation of stable SEI layers. PtTe_2_ maintains its layered structure during cycling and its basal planes facilitate the rapid surface diffusivity of Li. In contrast, PdTe_2_ and NiTe_2_ decompose to MTe_2‐_ *_x_* (M = Pd and Ni, 0 < *x* ≤ 2) forming Li_2_Te. The increased grain and interfacial boundary areas resulting from the phase decomposition provide additional nucleation sites, thereby reducing the diffusion path length of Li. The rapid surface diffusivity and increased number of nucleation sites dramatically reduces the crystallization overpotential, which is a component of *η*_n_. Moreover, Li_2_Te, a phase decomposition byproduct, contributes to the formation of a stable SEI layer, which reduces the interfacial energy between the electrolyte and deposited Li. The reduction in interfacial energy leads to a decrease in the critical size of Li nuclei. Thus, three factors are essential for improving the cycling performance of AFLBs and LMBs, namely, a high Li surface diffusivity on the CC and LMA, a large number of uniformly distributed nucleation sites, and the formation of a stable SEI.

## 2 Results and Discussion

### 2.1 AFLBs

**Figure 1:**
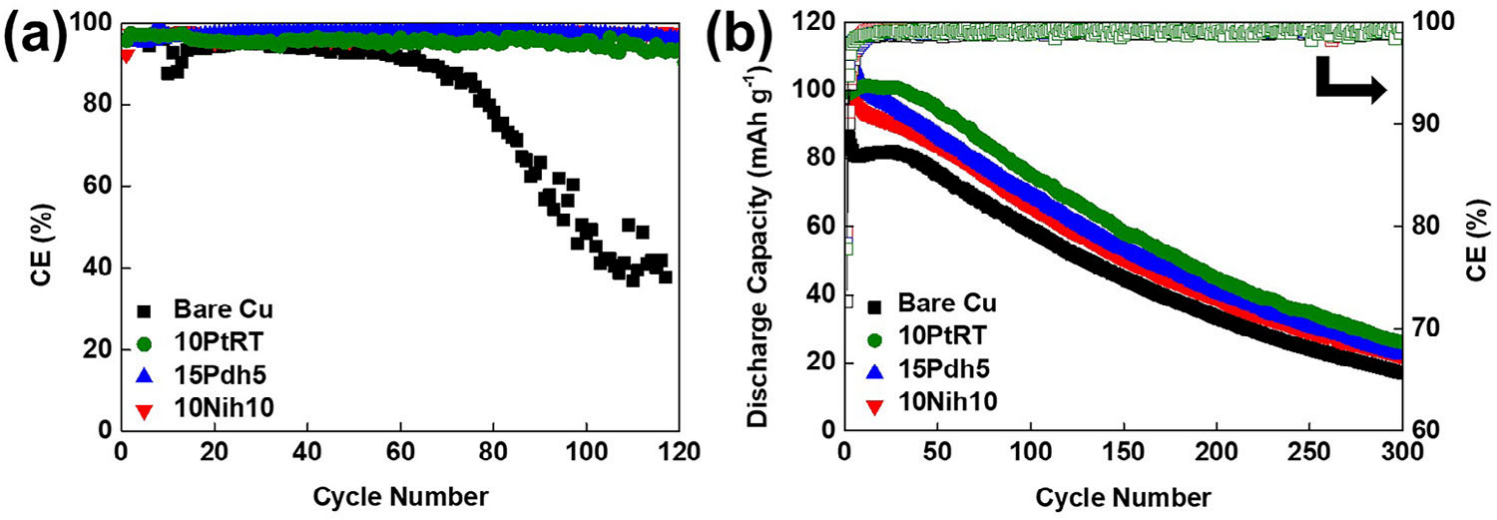
a) Coulombic efficiency (CE) of half‐cells containing CCs coated with 10‐nm‐thick PtTe_2_ without heating (10PtRT), 15‐nm‐thick PdTe_2_ with in situ heating at 473 K during sputtering and an additional 5 min heating (15Pdh5), and 10‐nm‐thick NiTe_2_ with in situ heating at 473 K during sputtering and an additional 10 min heating (10Nih10) tested at 1 mA cm^−2^ and 1 mAh cm^−2^. b) Cycling performance of full‐cells assembled with bare Cu, 10PtRT‐, 15Pdh5‐, and 10Nih10‐coated CCs and LiFePO_4_ (LFP) as a counter electrode (1.5 mAh cm^−2^) at a 0.2 C rate. Data shown are from the median‐performing cell among three independent cells (*n* = 3) per condition.

The PtTe_2_‐, PdTe_2_‐, and NiTe_2_‐coated CCs, as compared with the bare Cu CC, in half‐ (1 mA cm^−2^, 1 mAh cm^−2^) and full‐cells with LiFePO_4_ (LFP) as the counter electrode (1.5 mAh cm^−2^) exhibited superior performance at a 0.2 C rate, as shown in **Figure** **1**. Energy‐dispersive spectroscopy (EDS) using scanning electron microscopy (SEM) and atomic force microscopy (AFM) was employed to determine the compositional uniformity and thickness of the magnetron‐sputtered PtTe_2_, PdTe_2_, and NiTe_2_ thin‐films, as shown in Figures S1‒S4 (Supporting Information). In the half‐cell configuration, the PtTe_2_‐, PdTe_2_‐, and NiTe_2_‐coated CCs exhibited stable cycling performances for 120 cycles, whereas the CE of the bare Cu CC sharply decreased after ≈70 cycles. The 2D thin‐film fabrication process was meticulously optimized by adjusting both the film thickness and the duration of in situ annealing, as shown in Figure S5 (Supporting Information). The maximum CEs for CCs coated with PtTe_2_, PdTe_2_, and NiTe_2_ without heating were observed when the coating thickness was in the range of 5–15 nm. As shown in Figure S5 (Supporting Information), all three coatings exhibited a decrease in CE as the film thickness increased beyond this range, indicating that excessive thickness may hinder lithium nucleation and surface diffusion. Among the tested samples, the 15‐nm‐thick PdTe_2_ film subjected to in situ heating at 473 K during the sputtering process, followed by an additional 5 min of postdeposition annealing (identified as 15Pdh5), as compared with the other tested configurations, exhibited an enhanced cycling performance, as described in our previous study.^[^ ^10^ ^]^ Similarly, the 10‐nm‐thick NiTe_2_ film in situ heated at 473 K during sputtering and annealed for 10 min (identified as 10Nih10) and the 10‐nm‐thick PtTe_2_ film without in situ or post‐deposition heating (identified as 10PtRT) were selected for further investigation owing to their excellent performance. The average CEs of 15Pdh5 (97.6 ± 0.74%), 10Nih10 (97.1 ± 0.80%), and 10PtRT (95.5 ± 0.97%) for 120 cycles were considerably higher than that for the bare Cu CC (79.8 ± 19.78%), as shown in Figure 1a. The large standard deviation in CE for bare Cu is attributed to increasing electrical resistance during cycling. As shown in the voltage hysteresis profiles (Figure S6, Supporting Information), the voltage hysteresis of the bare Cu cell increases noticeably after ≈30 cycles, whereas the coated samples maintain more stable profiles. This indicates progressive resistance buildup in the bare Cu cell, likely due to the formation of an unstable SEI and possible dendritic growth. The rising resistance leads the cell to reach the cutoff voltage prematurely, resulting in incomplete Li stripping or plating and contributing to the observed CE variability. The coated CCs outperformed the bare CC (Figure 1b) in the full‐cell configuration. The first discharge capacities of the full‐cells with the coated CCs (PtTe_2_, PdTe_2_, and NiTe_2_ = 100.0, 110.8, and 112.4 mAh g^−1^, respectively) were higher than that of the bare Cu CC (94.2 mAh g^−1^). Therefore, coating the CC with the 2D materials improved the cycling performance in the half‐ and full‐cell configurations.

**Figure 2:**
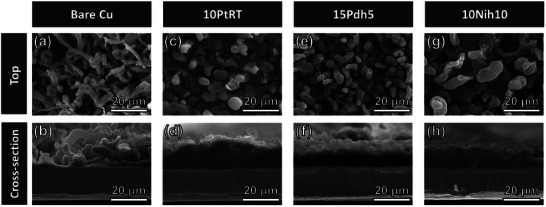
a,c,e,g) Top‐view and b,d,f,h) cross‐sectional SEM images of (a,b) the bare Cu and (c,d) 10PtRT‐, (e,f) 15Pdh5‐, and (g,h) 10Nih10‐coated CCs after the first plating of Li (1 mAh cm^−2^, 1 mA cm^−2^).

The superior cycling performance of the 2D material‐coated CCs is attributed to the uniform and dense deposition of Li, as shown in **Figure** **2**. Although the first Li plating on the bare Cu CC exhibited dendritic growth with a porous structure (Figure 2a,b), that on the 2D material‐coated CCs Li was evenly and densely deposited (Figure 2c–h). The uniform deposition of Li on the 2D material‐coated CCs remained consistent after 150 cycles, as shown in Figure S7 (Supporting Information).

**Figure 3:**
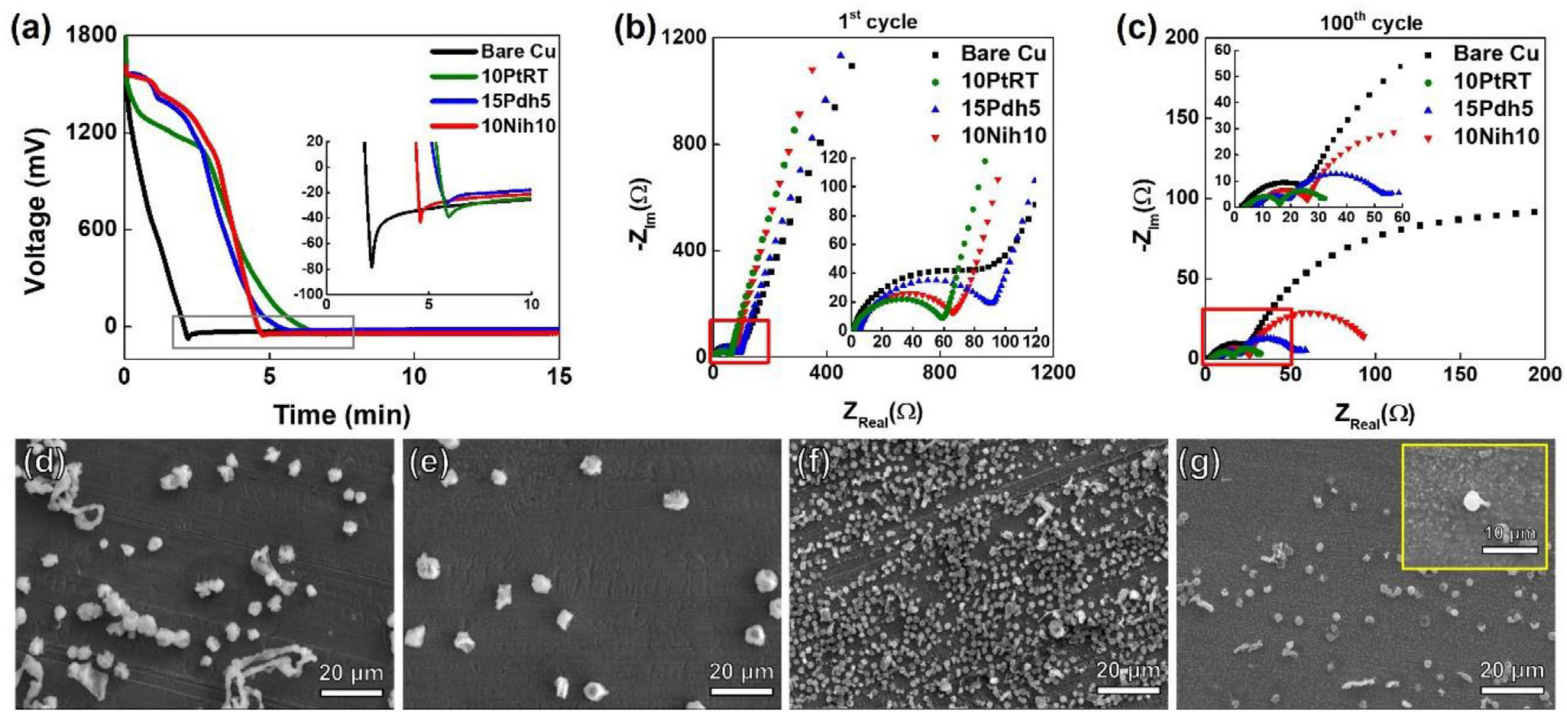
a) Overpotentials of the half‐cells with the 10PtRT‐, 15Pdh5‐, and 10Nih10‐coated and bare Cu CCs at 0.1 mA cm^−2^ and 0.5 mAh cm^−2^. Nyquist plots for the 10PtRT‐, 15Pdh5‐, and 10Nih10‐coated and bare Cu CCs (b) after the 1st and (c) 100th discharge cycles, as obtained from electrochemical impedance spectroscopy (EIS) measurements. The equivalent circuit diagram is provided in Figure S8 (Supporting Information). Top‐view SEM images of the (d) bare Cu and (e) 10PtRt‐, f) 15Pdh5‐, and (g) 10Nih10‐coated CCs after 30 min of Li plating (0.1 mA cm^−2^). Data shown are from the median‐performing cell out of three independent samples (*n* = 5) per condition.

The uniformity of Li deposition is determined by the nucleation behavior at the beginning of Li plating. Oyakhire et al. reported that the morphology of Li deposits is primarily determined by the *η_n_* on the coating materials.^[^ ^14^ ^]^ *η_n_* is the overvoltage (*η*) required to induce heterogeneous Li nucleation. The *η_n_* value is determined from the voltage‐plating time curve of AFLBs at the first cycle. This curve is characterized by an initial sharp spike, followed by a plateau, as shown in **Figure** **3**a. The absolute voltage value at the spike is *η_n_*. Since the *η* value depends on the resistances, the charge transfer resistance (*R*_ct_) and resistance to Li‐ion diffusion in the SEI layer (*R*_SEI_) were measured from Nyquist plot obtained via EIS measurements. The summarized EIS results in **Table** **1** clearly demonstrate the advantageous effects of the 2D‐material thin films on the *η_n_* value. First, the *η_n_* values on the 10PtRT‐, 15Pdh5‐, and 10Nih10‐coated CCs are lower than that on the bare CC (73.4 mV). Notably, the *η_n_* value on the 15Pdh5‐coated CC (16.5 mV) is approximately half that of the 10PtRT‐ (31.4 mV) and 10Nih10‐coated CCs (36.6 mV). Thus, it is expected that the 15Pdh5 induces more effective uniform Li deposition than 10PtRT, 10Nih10, and bare CC. Second, the half‐cell containing the 10PtRT‐coated CC exhibits a lower *R*_ct_ value (51.3 Ω) than the 15Pdh5‐ (110.8 Ω) and 10Nih10‐coated CCs (67.5 Ω) at the first cycle, as determined from the EIS measurements shown in Figure 3b. The *R*_ct_ and *R*_SEI_ values of all the 2D material‐coated samples reduced after 100 cycles, as compared to those measured in the first cycle, and they were lower than those of the bare CC (Figure 3c). Among the coated samples, the *R*_ct_ value of the 10PtRT‐coated CC remained lower than those of the 15Pdh5‐ and 10Nih10‐coated CCs, while the *R*_SEI_ values were comparable. The lower *R*_ct_ value observed for the 10PtRT‐coated sample can be attributed to the absence of phase decomposition during cycling, as comprehensively discussed below. Thus, the lower *η*_n_, *R*_ct_, and *R*_SEI_ values exhibited by the 2D material‐coated CCs demonstrate the improved uniformity of Li deposition on these coatings, as compared with that for the bare CC.

**Table 1:** *η*_n_ values determined from Figure 3a, and the SEI resistance (*R*_SEI_) and charge transfer resistance (*R*_ct_) at the 1st and 100th cycles obtained from the Nyquist plots in Figure 3b,c. *R*_n_ and Δ*V*_c_ were calculated using a current of 0.2 mA. All values are reported as mean ± standard deviation (SD) from five repeated measurements (*n* = 5) per condition.

Sample			Bare Cu	10PtRT	15Pdh5	10Nih10
*η*_n_ [mV]			73.4 ± 4.12	44.3 ± 3.83	28.8 ± 2.41	47.8 ± 4.70
EIS [Ω]	1st cycle	*R*_ct_	76.7 ± 2.84	51.3 ± 2.45	110.8 ± 3.98	67.5 ± 3.96
	100th cycle	*R*_SEI_	28.7 ± 9.26	14.0 ± 1.43	15.4 ± 1.64	22.5 ± 0.53
		*R*_ct_	163.9 ± 36.16	13.7 ± 1.62	51.8 ± 14.72	69.3 ± 4.92
*η*_c_			58.1	34.0	6.6	34.3

The effect of *η_n_* on Li plating was comprehensively examined through a numerical analysis. The lower *η_n_* value of the 15Pdh5‐coated sample, despite its higher *R_ct_* value, as compared with that of the 10PtRT‐coated sample, is attributed to a lower crystallization overpotential (*η_c_*), as demonstrated by the following calculation. Despite the co‐related kinetic processes that result in a blend of the four different *η* values within the voltage signal, the relative *η_c_* of the 2D thin films were isolated by using a previously reported method.^[^ ^15^ ^]^ *η_n_* is the sum of the *η*_c_, charge‐transfer overpotential (*η_ct_*), diffusion overpotential in the SEI layer (*η*_SEI_), and reaction overpotential (*η_r_*), as described in Equation (1):^[^ ^16^, ^17^ ^]^

(1) $$\eta_{n}=\eta_{c}+\eta_{\mathrm{ct}}+\eta_{\mathrm{SEI}}+\eta_{r}$$

*η_c_* was calculated using Equation (1) by subtracting *η*_ct_ from *η_n_*, while assuming negligible contributions from *η_SEI_* and *η_r_*. *η_ct_* was determined by multiplying the *R_ct_* value, measured during the first cycle in EIS, by the applied current (0.2 mA) used to measure *η_n_*.^[^ ^15^ ^]^ Notably, *η_SEI_* was considered to be negligible as it did not appear as a distinct arc in the Nyquist plots at the first cycle (Figure 3b). *η_r_* was assumed to be minimal based on the following considerations: (1) both 15Pdh5 and 10Nih10 undergo identical decomposition reactions under identical electrochemical environments, namely, MTe_2_ → MTe_2−_ *_x_* + Li_2_Te, where M = Pd or Ni (0 ≤ *x* ≤ 2), suggesting comparable *η_r_* values. Despite the comparable *η_r_* values, the notable differences in the *η_n_−η_ct_* (i.e., *η_c_* +*η_r_*) values between 15Pdh5 (6.6 mV) and 10Nih10 (34.3 mV) indicate that *η_c_*, rather than *η_r_*, is the predominant factor; and (2) the low *η_c_* +*η_r_* value for 15Pdh5 indicates an upper limit for *η_r_* (<6.6 mV), supporting its insignificance. Thus, the *η_c_* value was calculated in this study by subtracting *η_ct_* from *η_n_*. All the 2D material‐coated CCs exhibited lower *η_c_* values than the bare CC, as summarized in Table 1. In particular, the 15Pdh5‐coated CC was more effective in reducing the *η_c_* value than the 10PtRT‐ or 10Nih10‐coated CCs.

The numerical comparison of *η_c_*, discussed earlier, is further supported by the nucleation and growth behavior at the onset of Li plating. *η_c_* is a function of the surface diffusivity and diffusion length of Li adatoms on the CC to the nucleation sites.^[^ ^16^ ^]^ Shorter diffusion lengths (i.e., a higher number of evenly distributed nuclei) and/or higher diffusivity decrease *η_c_*. Considering the number of nuclei in the SEM images of the Li deposited after the first 30 min of plating at 0.1 mA cm^−2^ (Figure 3d–g), 15Pdh5 exhibits a larger number of smaller and evenly distributed Li islands than 10PtRT and bare Cu. The larger quantity of Li islands on 15Pdh5 shortens the diffusion length for Li atoms, supporting its lower *η_c_* value. The average distance between two adjacent Li islands (2*x*_0_) on 15Pdh5 is ≈3.3 ± 0.9 µm, which is significantly shorter than the 21.9 ± 7.7 and 12.4 ± 4.3 µm on 10PtRT and the bare Cu, respectively, as listed in **Table** **2**. The 10Nih10 sample exhibited two 2*x*_0_ values, namely, 0.5 ± 0.1 and 4.4 ± 1.8 µm, corresponding to two distinct types of Li islands, that is, Li islands with ≈500 nm and 4 µm diameters, respectively. The reason for the higher *η_c_* value of 10Nih10, as compared with that of 15Pdh5, despite the shorter 2*x*_0_ on 10Nih10, is further discussed below.

**Table 2:** Diffusivity of Li on bare Cu, 10PtRt, 15Pdh5, and 10Nih10 calculated using Equation (4) and *D = L* ^2^/*t*, where *L* is half of the radius of the island area. In the case of 10Nih10, only larger (smaller) nuclei were considered for the estimation. Island diameter and inter‐island spacing were determined from 100 particles (*n* = 100) and 100 inter‐island distances (*n* = 100), respectively, based on SEM images. All values are reported as mean ± SD.

		Bare Cu	10PtRT	15Pdh5	10Nih10
Li island diameter [µm]		4.8 ± 0.8	7.3 ± 1.2	2.3 ± 0.6	0.5 ± 0.1 (2.5 ± 0.6)
2*x*_0_ [µm]		12.4 ± 4.3	21.9 ± 7.7	3.3 ± 0.9	0.5 ± 0.1 (4.4 ± 1.8)
Number of Li atoms per island		1.3 × 10^12^	4.7 × 10^12^	1.5 × 10^11^	1.5 × 10^9^
Number of electrons in the area		1.4 × 10^12^	4.3 × 10^12^	9.6 × 10^10^	2.2 × 10^9^
Diffusivity [m^2^ s^−1^]	Equation (4)	2.4 × 10^−14^	8.3 × 10^−14^	4.4 × 10^−15^	6.9 × 10^−17^
	Equation (5)	2.7 × 10^−15^	8.4 × 10^−15^	1.9 × 10^−16^	1.0 × 10^−17^

Diffusivity, in addition to the diffusion length, should also be considered when discussing *η_c_*. The surface diffusivities on the 2D material coatings were numerically estimated using the Li island growth behavior and calculated *η_c_* value. The areal concentration of adatoms (mol m^−2^) at a large distance from a growth site (*c_∞_*) can be described using Equation (2):^[^ ^16^ ^]^

(2) $$c_{\infty }=\bar{c}\left\{\exp\left(-\frac{zF}{RT}\eta_{c}\right)\right\}$$

where $\bar{c}$, *z*, *F*, *R*, and *T* are the areal concentrations of the adatoms at the nucleation site, charge‐transfer valences, Faraday's constant, gas constant, and temperature, respectively. The average current density (*i*) between the two growth sites separated by 2*x*_0_ can be described by Equation (3):^[^ ^16^ ^]^

(3) $$i=-\frac{zFD}{x_{0}}\cdot {\left(\frac{\partial c}{\partial x}\right)}_{x=0}$$

Equation (4) can be derived from Equations (2) and (3) as:

(4) $$i=\frac{zFDC_{\infty }}{{x_{0}}^{2}}\left\{1-\exp\left(\frac{zF}{RT}\eta_{c}\right)\right\}$$

The diffusivity under the constant current condition in which the *η* value was measured can be calculated by inputting the *η_c_* and *x*_0_ values into Equation (4). For example, the *D* value for the bare CC sample can be calculated as follows: (1) the average Li island diameter on the bare CC, as shown in Figure 3, is 4.8 µm; (2) under the assumption that each island is hemispherical, this corresponds to 2.2 × 10^−12^ mols of Li or ≈1.3 × 10^12^ Li atoms; and (3) the number of electrons transferred over a circular area with a diameter of 2*x*_0_, 15 µm, for 30 min at 0.1 mA cm^−2^ is 1.4 × 10^12^ (Table 2), which corresponds with the number of Li atoms in each Li island. This confirms that Li islands were formed by the radial diffusion of Li ions within the circular area. The *c_∞_* value was calculated by dividing the amount of Li (2.2 × 10^−12^ mols) by the circular area, which results in *c_∞_* = 0.04 mol m^−2^. Using the *η_c_* and the 2*x*_0_ values in Tables 1 and 2, the diffusivities of the Li adatoms on the CCs were calculated using Equation (4). The diffusivities were compared with those obtained by using the diffusion Equation (5):

(5) $$L=\sqrt{2Dt}$$

where *L* is the diffusion length and *t* is the time. The calculation was performed by using *x*_0_/2 as *L* and a plating time (*t*) of 30 min because the Li ions landing at any point within a circular area of radius *x*_0_ diffuse toward the center of the circle. Consequently, the average diffusion length of a landed Li ion is *x*_0_/2.

Similarly, the diffusivities of the Li adatoms on the 2D thin films were calculated using the above procedure, with the exception of the 10Nih10‐coated CC. Two distinct types of Li islands were observed on this CC, namely, initial small islands that almost touched each other and larger islands growing on top of the smaller islands. The larger islands likely formed through the diffusion of Li via the predeposited small Li islands. The self‐diffusivity of Li is ≈10^−14^ m^2^ s^−1^.^[^ ^18^, ^19^ ^]^ Assuming this self‐diffusivity, large Li islands would require ≈1050 s to grow on top of the predeposited Li, as calculated using Equation (5). Accordingly, the growth of smaller islands on 10Nih10 requires ≈750 s, with a total growth time of 1800 s. This growth time, along with *x*_0_/2 and *η_c_*, were used to calculate the Li adatom surface diffusivity on 10Nih10 during small island growth using Equations (4) and (5). Table 2 summarizes the *D* values calculated using Equations (4) and (5). The relative values of all surface diffusivities calculated using Equations (4) and (5) remained consistent, whereas those calculated Equation (4) were approximately an order of magnitude higher than those calculated using Equation (5).

The diffusivity of 10PtRT was approximately threefold higher than that of bare Cu, clearly confirming that the lower *η_c_* on 10PtRT originates from enhanced surface diffusion, even though the 2*x*_0_ was longer. This improvement in diffusivity is directly linked to the thermodynamic and crystallographic stability of the PtTe_2_ phase. Specifically, X‐ray diffraction (XRD) (Figures S9 and S10 and Table S1, Supporting Information) and scanning spreading resistance microscopy (SSRM) (Figure S11, Supporting Information) analyses revealed that the PtTe_2_ coating retained its 2D‐layered structure with a high proportion of grains oriented along the (001) plane parallel to the substrate (001‐grains). Approximately 70% of the coating area was covered with 001‐grains, which provide an environment for rapid surface diffusion. This basal‐plane orientation provides a low‐energy diffusion pathway due to the absence of dangling bonds, facilitating rapid Li surface diffusion.^[^ ^11^, ^20^, ^21^, ^22^ ^]^ In contrast, PdTe_2_ and NiTe_2_ coatings undergo phase decomposition during cycling, as evidenced by the emergence of PdTe and NiTe phases. This decomposition reduces the long‐range crystallographic order and introduces defects that disrupt surface diffusion, thereby lowering diffusivity. However, the accompanying increase in grain boundaries and interfacial areas creates numerous thermodynamically favorable nucleation sites, as shown in Figure 3f,g. Consequently, although the diffusivity is reduced, the nucleation overpotential remains low due to a shorter diffusion path. Thus, phase stability determines whether enhanced diffusivity (as in PtTe_2_) or increased nucleation site density (as in PdTe_2_ and NiTe_2_) dominates the overall Li plating behavior.

The differing reactivity of PtTe_2_, PdTe_2_, and NiTe_2_ with lithium during deposition can be understood by analyzing their respective reaction mechanisms, which are directly governed by the phase stability of these 2D materials under electrochemical conditions. The basal plane of the 2D phase facilitates the rapid surface diffusivity of Li, whereas the phase decomposition is expected to increase the number of nucleation sites owing to the expansion of the grain boundary and interfacial areas. The general formula for TMDs is MX_2_ (M = transition element, X = chalcogen species). In addition to the MX_2_ phase, M‐X compounds can exist as other intermetallic phases with compositions that deviate from the 1:2 ratio, such as MX_2−_ *_x_* (0 < *x* < 2, e.g., MX and M_2_X_3_). The relative formation energy of MX_2_ during Li intercalation is expected to govern its phase stability. When the formation energy of MX_2_ is similar to or higher than that of the MX_2−_ *_x_* phases, the phases are likely to decompose into MX_2−_ *_x_* forming LiX*_y_*. This expectation corresponds with previous findings. Price et al. employed first‐principles calculations to demonstrate that SnS_2_ and PdS_2_ readily decompose into MX_2−_ *_x_* forming LiX*_y_* upon Li intercalation, whereas NiS_2_, ZrS_2_, GeS_2_, SnSe_2_, and MoS_2_ retain their layered structures.^[^ ^23^ ^]^ The trend in the relative formation energies between the MX_2_ and MX_2−_ *_x_* phases, calculated using the Materials Project (materialsproject.org), is consistent with the findings reported by Price et al.^[^ ^24^, ^25^ ^]^ That is, the NiS_2_, ZrS_2_, GeS_2_, SnSe_2_, and MoS_2_ formation energies are lower than those of their MX_2−_ *_x_* phases. Based on these trends and our experimental data, the reaction mechanisms can be summarized as follows: PtTe_2_ remains chemically inert during lithium exposure, maintaining its layered structure without forming secondary phases. In contrast, PdTe_2_ and NiTe_2_ undergo conversion‐type reactions, forming PdTe/NiTe and Li_2_Te. These can be described by the simplified reaction equation:

(6) $$\mathrm{MT}e_{2}+2\mathrm{xLi}\to \mathrm{MT}e_{2-x}+\mathrm{xL}i_{2}\mathrm{Te}\left(M=\mathrm{Pd},\;\mathrm{Ni}\right)$$

**Figure 4:**
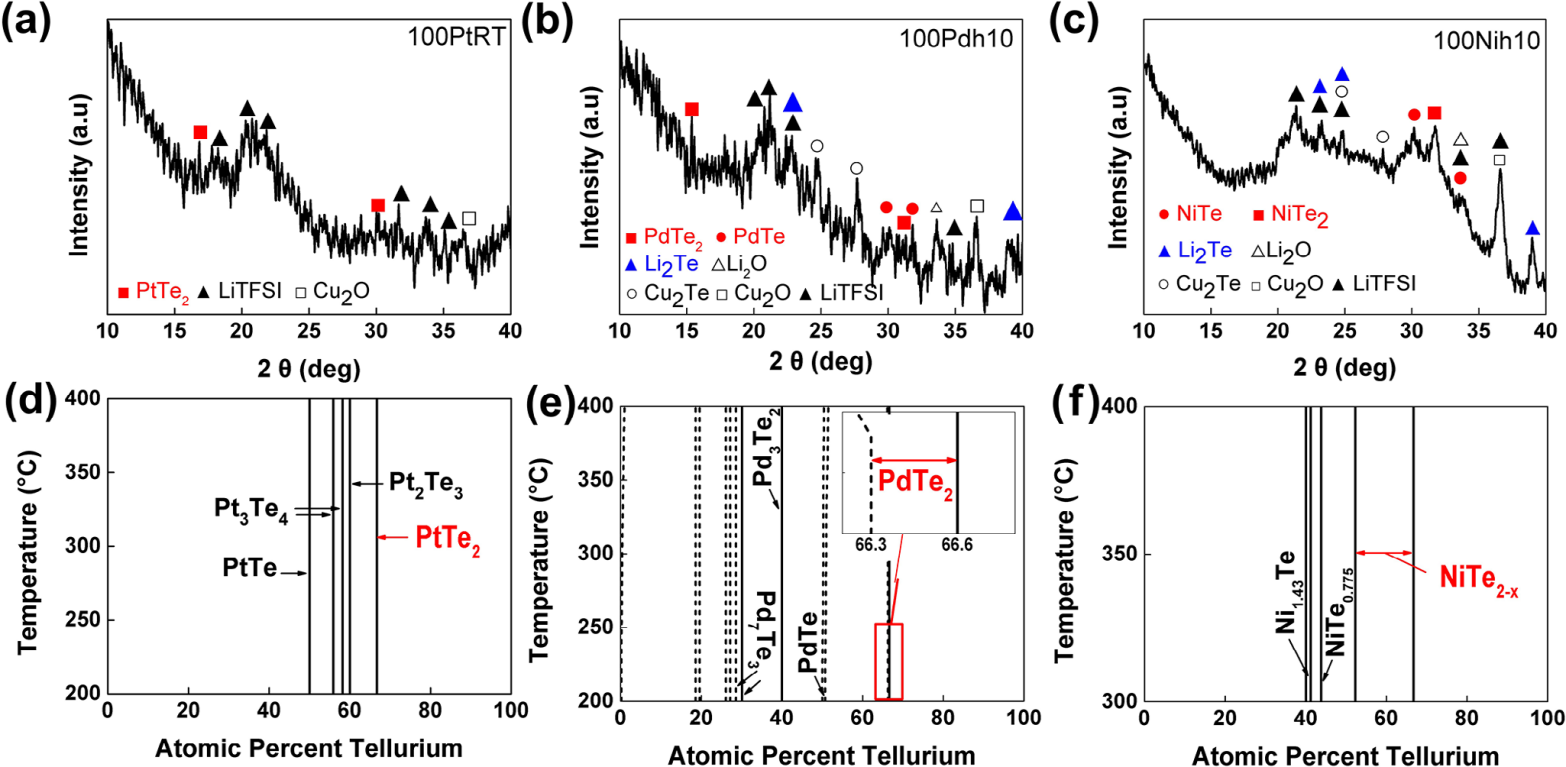
XRD analyses of the CC coated with 100‐nm‐thick a) 100PtRT, b) 100Pdh5, and c) 100Nih10 thin films after 0.5 cycles in the half‐cell configuration (1.0 mAh cm^−2^ @ 0.1 mA cm^−2^). The 100‐nm‐thick coating was applied to carefully analyze the phase transition in the 2D thin films. d) Pt‒Te, e) Pd‒Te, and f) Ni‒Te phase diagrams, reprinted with permission from T. B. Massalski et al., Binary Alloy Phase Diagrams, 2nd ed., ASM International, 1986. Copyright 1986 ASM International. All rights reserved.

Our experimental observations support this mechanism. The XRD analysis showed that PdTe_2_ decomposed into PdTe_2−_ *_x_* forming Li_2_Te upon Li deposition, while PtTe_2_ maintained its layered structure after 0.5 cycle (**Figure** **4**a). To conduct accurate XRD analyses of the phase transition, 100‐nm‐thick PdTe_2_, NiTe_2_, and PtTe_2_ thin films were fabricated. In addition to the early‐stage (0.5 cycle) analysis, we performed post‐cycling XRD after 50 cycles to assess the long‐term stability of the coatings (Figure S12, Supporting Information). PtTe_2_ retained its layered structure, showing no signs of phase decomposition, while NiTe_2_ partially transformed into NiTe, similar to what was observed after 0.5 cycle. In contrast, PdTe_2_ was no longer detectable, and peaks corresponding to PdTe became more prominent, indicating that PdTe_2_ underwent complete phase transformation during cycling. Consistent with the mechanism described above, the XRD analysis (Figure 4a and Figure S12, Supporting Information) confirms that the layered structure of PtTe_2_ (−0.442 eV/atom) remained intact during Li deposition, showing no signs of decomposition into PtTe (−0.401 eV/atom). This thermodynamic stability reflects PtTe_2_’s lower formation energy, which renders it more resistant to reduction by lithium compared to PdTe_2_ and NiTe_2_. In contrast, despite having a lower formation energy (−0.225 eV/atom) than NiTe (−0.190 eV/atom), NiTe_2_ partially decomposed after cycling, as confirmed by XRD analysis showing the presence of NiTe_2_, NiTe, and Li_2_Te (Figure 4c and Figure S12, Supporting Information). This apparent instability can be attributed to the nonstoichiometric nature of NiTe_2_, which has a broad Te composition range that promotes Te vacancy formation and structural disorder under lithium exposure. Stoichiometric intermetallic compounds (SCs) and nonstoichiometric intermetallic compounds (nSCs) can be distinguished using their phase diagrams,^[^ ^26^ ^]^ as illustrated in Figure 4d‒f. A SC, as depicted for PtTe_2_, is represented as a single vertical line, whereas nSC is depicted as an area with a specific Te content range, such as PdTe_2−_ *_x_* (0 ≤ *x* ≤ 0.03) and NiTe_2−_ *_y_* (0 ≤ *y* ≤ 0.9). The relatively large Te content range in NiTe_2_ facilitates the release of Te atoms, resulting in the formation of Te vacancies in NiTe_2_ and the subsequent formation of Li_2_Te. The creation of these vacancies disrupts the long‐range crystallographic order of NiTe_2_ and leads to partial decomposition of NiTe_2_ into NiTe while some regions remain as NiTe_2_. The disruption of the long‐range order causes the (001) peak in the XRD profile at 16.7° to disappear, whereas the (002) peak at 33.9° remains visible, as shown in Figure 4c. The instability of the 2D phase was also confirmed by the formation of Cu‒Te phases between the Cu and 10Nih10 or 15Pdh5 CC films, whereas no Cu‒Te phases were formed at the interface of the 10PtRT and Cu CC films (Figure S11, Supporting Information). This suggests that the instability of the MTe_2_ 2D phase at the interface with Li is primarily determined by the relative formation energies of the corresponding MTe*_x_* (0 < *x* < 2) phases and is further influenced by their nonstoichiometric characteristics.

In addition to surface diffusivity and the number of nucleation sites, the byproduct of phase decomposition, Li_2_Te, also plays a significant role in influencing the nucleation behavior. Despite the comparable *η_n_* values between 10PtRT and 10Nih10, the greater number of smaller Li islands observed on 10Nih10 (see Figure 3) is attributed to the larger number of nucleation sites created by the phase decomposition of 10Nih10. Furthermore, the interfacial energy between the deposited Li and electrolyte (*γ_NE_*) must also be considered, as *γ_NE_* is a critical factor in determining the critical nucleus radius. Ely and García stated that the critical radius required to form thermodynamically and kinetically stable nuclei can be determined using Equations (7) and (8), respectively:^[^ ^27^ ^]^

(7) $$\;r_{eq}^{*}=\frac{2\gamma_{NE}\Omega }{zF\eta +\Delta G_{f}\Omega }\;$$

(8) $$\;r_{k}^{*}=\frac{2\gamma_{NE}\Omega }{zF\eta }\;$$

where Ω and Δ*G*_f_ are the molar volume of Li and volume free energy of transformation, respectively. r^*^_k_ is larger than r^*^_eq_, and the time required for embryos to grow from r^*^_eq_ to r^*^_k_ defines the incubation time.^[^ ^27^ ^]^ When *η* is sufficiently large, r^*^_eq_ and r^*^_k_ become comparable, allowing nuclei to form without an incubation period. Conversely, smaller *η* values increase the deviation between r^*^_eq_ and r^*^_k_, necessitating an incubation time for nucleation. Under comparable *η_n_* values for 10Nih10 and 10PtRT, the more than order‐of‐magnitude smaller diameter of Li islands observed on 10Nih10, as compared with those on 10PtRT, can be attributed to the lower *γ_NE_* value between the electrolyte and Li deposited on 10Nih10. This reduction in *γ_NE_* is due to the stable SEI layer formed by the lithiophilic Li_2_Te generated during the decomposition of NiTe_2_,^[^ ^28^ ^]^ as previously discussed. Therefore, numerous nucleation sites, a high Li surface diffusivity on the CC, and a reduction in *γ_NE_* by the formation of a stable SEI layer are essential for uniform Li plating on the CC in AFLBs. Uniform Li plating minimizes the total surface area of Li deposits. This reduced surface area per unit volume of deposited Li significantly decreases the consumption of electrolyte by limiting the formation of an SEI, thereby enhancing battery performance.^[^ ^14^ ^]^

### 2.2 LMBs

**Figure 5:**
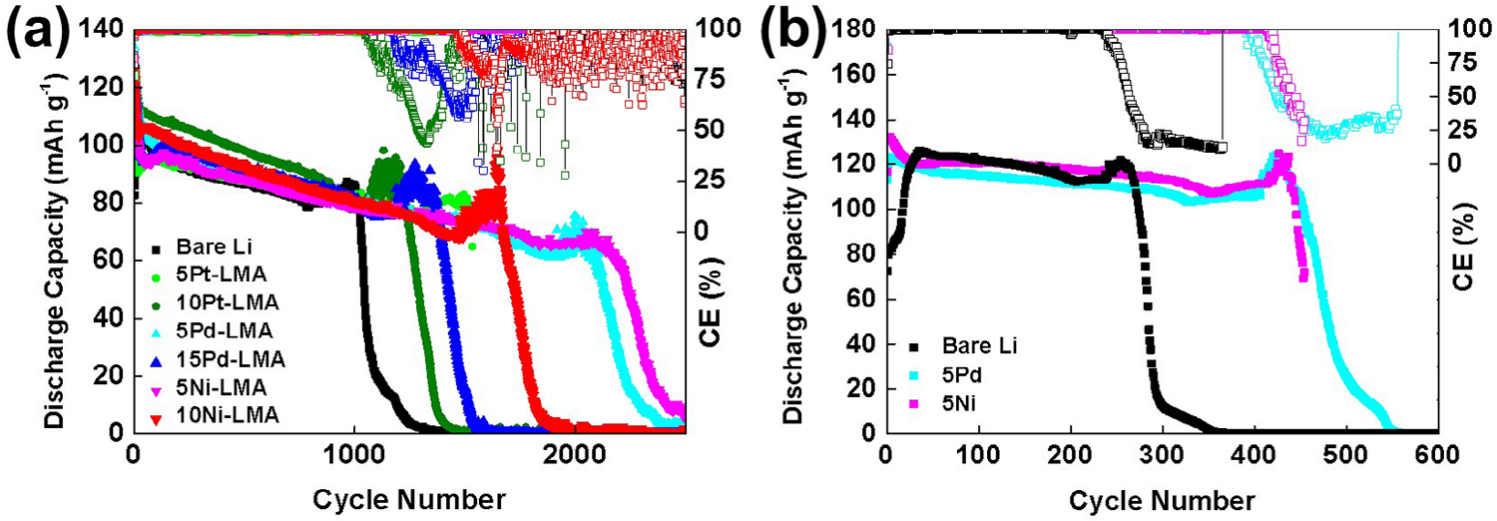
Cycling performance of full‐cells assembled with a) 5‐ and 15‐nm‐thick PdTe_2_‐, 5‐ and 10‐nm‐thick NiTe_2_‐, and 5‐ and 10‐nm‐thick PtTe_2_‐coated LMAs and LFPs as the counter electrodes (1.5 mAh cm^−1^) at a 1 C rate, as compared with a full‐cell with a bare LMA, and (b) with 5‐nm‐thick PdTe_2_‐ and NiTe_2_‐cotaed LMAs and LFPs as the counter electrodes with 3.0 mAh cm^−1^ at a 0.5 C rate.

LMAs were also coated with the 2D thin films, which significantly improved the cycling performance of the full‐cells, as compared with that of bare Li anodes. Because of the low melting temperature of Li, in situ heating during thin‐film deposition was not applied, and the cycling performance of the coated LMA was optimized only by varying the coating thickness, as shown in **Figure** **5**. Among the 2D coatings with various thicknesses, the LMAs with 5‐nm‐thick 2D coatings exhibited a longer cycling life than bare Li anodes and those with 10‐ or 15‐nm‐thick 2D coatings, which led to the maximum cycling performance in AFLBs. Among the 5‐nm‐thick 2D coatings, the LMAs with the PdTe_2_ (5Pd) and NiTe_2_ (5Ni) coatings exhibited higher capacity retention and extended cycling life than that with 5PtTe_2_ (5Pt) in the full‐cell configuration with the LFP cathode (1.5 mAh cm^−2^), as shown in Figure 5. This may indicate that the formation of a stable SEI layer containing Li_2_Te with numerous nucleation sites resulting from the decomposition of 5Pd and 5Ni is more critical than the high surface diffusivity (exerted by 5Pt) for the improved cycling performance of LMB.

**Figure 6:**
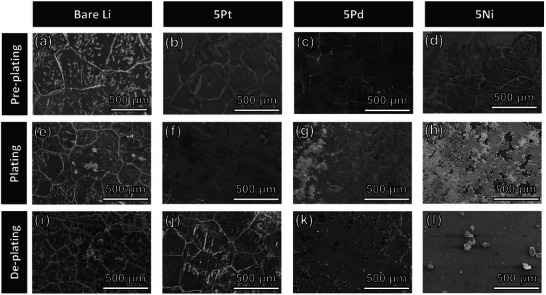
SEM images of the surface morphologies of (a,e,i) bare Li metal foil, b,f,j) 5Ni, c,g,k) 5Pd, and (d,h,l) 5Pt after (a–d) 24 h immersion in the electrolyte, (e–h) 1st plating, and (i–l) 1st stripping.

The importance of the coating on the LMA and the formation of a stable SEI layer were confirmed by analyzing the LMA surface morphology, both prior to plating and after the first plating and stripping cycles, as shown in **Figure** **6**. Before cycling, immersion in the electrolyte for 24 h with no applied voltage led to the formation of etch pits on the surface of the bare Li metal foil (Figure 6a), as compared with the as‐received Li foil (Figure S13, Supporting Information). The etch pits formed along the grain boundaries and within the grains, leading to uneven surface conditions that resulted in poor cycling performance. In contrast, no etch pits formed on 5Pt (Figure 6b). The stable PtTe_2_ thin film, which exhibited no phase decomposition in the AFLBs, effectively protected the Li metal from attack by the electrolyte. Instead of forming etch pits, ≈100‐nm‐diameter dots were primarily formed along the grain boundaries of 5Pt. Etch pits were formed on 5Ni and 5Pd, but their quantity and sizes were considerably smaller than those on bare Li (Figure 6c,d). After the first plating, etch pits and cracks along the grain boundaries remained on the bare Li foil (Figure 6e), whereas no etch pits were observed on the 2D‐coated LMAs. The plated Li effectively covered the etch pits, as shown in Figure 6f–h. After the first stripping, etch pits and cracks along the grain boundaries reappeared on the bare Li (Figure 6i) and on 5Pt (Figure 6j), respectively. However, few etch pits and cracks were observed, and the grains boundaries on 5Ni and 5Pd were almost indistinguishable (Figure 6k,l). The number of available nucleation sites and the formation of a stable SEI layer during the first plating, as confirmed in the AFLBs, may lead to uniform plating and stripping of the LMBs. Thus, uniform plating and stripping resulted in a better cycling performance. Full‐cells containing 5Ni‐ or 5Pd‐coated LMA and LFP cathodes with a commercial‐level areal capacity (3 mAh cm^−2^) were evaluated, as shown in Figure 5b. The full‐cells containing the 5Ni‐ and 5Pd‐coated LMAs demonstrated capacity retentions exceeding 80% after 440 and 450 cycles, respectively. In contrast, the bare Li anode exhibited a capacity retention exceeding 80% after 270 cycles. The full‐cells containing the 5Ni and 5Pd coatings fabricated in this study outperformed previously reported full‐cells containing LMAs improved through coating strategies. As summarized in Table S3 (Supporting Information), this performance surpasses that of previous studies including refs. [29, 30] and^[^ ^31^ ^]^ which either demonstrated lower retention or fewer cycles under similar or lower areal capacities. Notably, ref. [31] achieved 66.6% after 1500 cycles, but at only 1.5 mAh cm^−2^. Our result demonstrates one of the highest reported stabilities at a commercial‐level areal capacity.

## 3 Conclusion

This study highlights the relevance of 2D coatings in enhancing the performance of next‐generation batteries by providing insights into the fundamental mechanisms underlying Li nucleation and diffusion. The 2D thin‐film coatings on the CC in AFLBs and on the LMA in LMBs significantly improved their cycling performance. The 2D coatings in AFLBs reduced the *η_n_* value, which is a key factor determining the uniformity of deposited Li. The reduction in *η_n_* was attributed to a decrease in the *η_c_* value, which is governed by the surface diffusivity and diffusion path length of Li adatoms on the CC. Rapid surface diffusivity and shorter diffusion paths effectively reduced the *η_c_* value. The PtTe_2_ coating enhanced surface diffusivity, whereas the PdTe_2_ and NiTe_2_ coatings provided a greater number of nucleation sites, thereby shortening the diffusion path of the Li adatoms. These differences in contribution to the reduction in the *η_c_* value originated from the phase stability of the coatings. PtTe_2_, which is more stable than other Pt‒Te intermetallic compounds, remained intact with its basal plane aligned parallel to the substrate, facilitating rapid Li surface diffusivity. In contrast, PdTe_2_ decomposed into a more stable phase, PdTe, while forming Li_2_Te. The phase transition increased the number of nucleation sites despite a reduction in surface diffusivity. Similarly, NiTe_2_ decomposed into NiTe forming Li_2_Te, providing additional nucleation sites, although NiTe_2_ is more stable than NiTe. The NiTe_2_ phase transition is attributed to its nonstoichiometric characteristics. In addition to the surface diffusivity and availability of nucleation sites, the interfacial energy between the electrolyte and Li nuclei governs the nucleation behavior. Lithiophilic Li_2_Te, formed as a byproduct of the decomposition of PdTe_2_ and NiTe_2_, contributes to the formation of a stable SEI layer, reducing the interfacial energy between the electrolyte and Li nuclei. The reduced interfacial energy lowers the critical nucleus size, thereby enabling a larger number of nuclei to continue to grow. The PdTe_2_‐ and NiTe_2_‐coated LMAs in the LMBs exhibited superior cycling performances, as compared with both the bare and PtTe_2_‐coated LMAs. Therefore, this observation highlights that the availability of nucleation sites and the formation of a stable SEI layer are more critical for improving the LMB performance than rapid surface diffusivity. Furthermore, this trade‐off between diffusivity and nucleation density is a key design consideration for optimizing interfacial engineering in high‐performance lithium‐metal and anode‐free batteries.

## 4 Experimental Section

### Manufacture of the PtTe_2_, PdTe_2_, and NiTe_2_ Sputtering Targets

PtTe_2_, PdTe_2_, and NiTe_2_ sputtering targets were produced using the methodology detailed in previous study.^[^ ^32^, ^33^ ^]^ The metallic elements (M = Pt, Pd, and Ni) and Te were weighed based on an atomic ratio of M:Te = 33:67. This slightly Te‐rich composition was chosen to compensate for the potential loss of Te during high‐temperature solid‐state synthesis, as Te has a high vapor pressure and can volatilize under heating. This adjustment ensures the formation of stoichiometric MTe_2_ phases, as confirmed by XRD analysis (Figure S1, Supporting Information).

These elements were placed in a vacuum‐sealed quartz tube and heated at 1273 K for 24 h to facilitate the formation of PtTe_2_, PdTe_2_, and NiTe_2_ intermetallic compounds. The resulting intermetallics were gradually cooled to 773 K at 373 K h^−1^, and subsequently to 293 K in the furnace. Subsequently, PtTe_2_, PdTe_2_, and NiTe_2_ were pulverized into powdered forms and sintered to create sputtering targets using spark plasma sintering (Fuji Electronic Industrial Co., Ltd., SPS‐630Lx). Sintering was conducted at 653 K for 40 min at a pressure of ≈2 kPa in an Ar atmosphere.

### Preparation of the Coated Copper CCs and LMAs

Using the prepared PtTe_2_, PdTe_2_, and NiTe_2_ targets, PtTe_2_, PdTe_2_, and NiTe_2_ thin films were deposited onto both Cu (18‐µm‐thick, UACJ) and Li foils (200‐µm‐thick, Honjo Metal Co., Ltd.) using a magnetron sputtering system (base pressure: 4 × 10^−6^ Torr, working pressure: 5 × 10^−3^ Torr, using Ar (99.999%; Gaschem Technology)) with a DC power of 40 W. The Cu foil was sputtered with or without heating at 473 K. Subsequently, the coated Cu CC was maintained in the vacuum sputtering chamber until its temperature reached 300 K. Following the sputtering process, the coated Cu CCs and LMAs were directly transferred to a glovebox through a gate to prevent exposure to ambient air to prepare the cells.

### Characterization

SEM (JEOL Ltd., JSM‐6510LA) was used to analyze the surface and cross‐sectional morphologies of the coated Cu CCs and LMAs. The SEM analysis samples were prepared in an Ar‐filled glovebox. The samples were rinsed with 1,2‐dimethoxyethane (DME; Sigma Aldrich) to eliminate any residual Li salts present in the glovebox. Subsequently, the samples were hermetically sealed in a container to minimize the exposure time to ambient air during the SEM transfer and loading process. EDS (Energy Oxford, x‐Act 6) was used to generate elemental mapping images of the samples. The crystal structures of the samples were analyzed using grazing incidence XRD (Panalytical B.V., EMPYREAN) with a Cu K𝛼 source, and the diffraction patterns were matched via High Score Software. The electronic conductivity was measured on the top surface of the 2D‐coated CCs using the SSRM mode of AFM (XE‐200, Park Systems). The SSRM mode can be operated in the range of 10 pA to 100 mA. The current was measured in contact mode using diamond‐coated tips (CDT‐NCHR, Park Systems). The current flowing from the tip to the sample base at a bias of 3 V was measured using a logarithmic current amplifier in the SSRM mode. Topographic and current images were simultaneously acquired during each single scan. AFM was also employed to determine the thickness of the thin films deposited on SiO_2_/Si wafers (≈300‐nm‐thick SiO_2_ wafers; Namkang Hi‐Tech Co., Ltd.). The contact mode was applied, and the set point was maintained at 50 nN. Gwyddion software was employed to visualize the conductivity, topography, and thickness of the coatings.

### Cell Assembly and Electrochemical Measurements

Half‐ and full‐cell tests were conducted using CR 2032‐coin cells. The performances of the cells were assessed using a battery test system (Wonatech, WBCS 3000Le) at room temperature following a 24‐h aging period. The cells were assembled in an Ar‐filled glovebox (O_2_ < 1 ppm, H_2_O < 1 ppm). The electrolyte employed in the cells was Li bis(trifluoromethanesulfonyl)imide (1 m; Dongwha Electrolyte) in a 1:1 ratio of 1,3‐dioxolane/DME (Dongwha Electrolyte) with 2 wt% Li nitrate (70 µL, Alfa Aesar). Celgard 2400 was used as a separator. The Cu and coated Cu CCs were cut into 18‐mm‐diameter rounds, while the Li metal foil for the half‐cells was cut into 16‐mm‐diameter rounds. The half‐cell cycling tests were conducted at 1.0 mA cm^−2^ and 1.0 mAh cm^−2^. Impedance measurements were performed using EIS using a potentiostat/galvanostat (Wonatech, Zive SP2) within the frequency ranges of 1 MHz to 100 mHz (in the AFLBs) and 1 MHz to 10 mHz (in the LMBs), with an amplitude of 5 mV at room temperature. ZMAN software was used to fit the EIS data.

LFP cathodic active materials (1.0, 1.5, and 3.0 mAh cm^−2^) were employed for the full‐cell tests. The cathode was prepared by creating a slurry with a LFP (Welcos):carbon black (Sigma Aldrich):binder (poly‐1,1‐difluoroethene (Alfa Aesar):1‐methyl‐2‐pyrrolidone (Sigma Aldrich) = 1:24) weight ratio of 8:1:1. This mixture was coated on an Al foil (15‐µm‐thick, UACJ), vacuum dried, and rolled. The cathode was then cut into 14‐mm‐diameter rounds. The Cu and coated Cu CCs and LMAs were cut into 16‐mm‐diameter rounds for the full‐cells. The full‐cells were cycled at 0.2, 0.5, and 1.0 C with a voltage window of 3.0−3.8 V. The average CEs of the half‐cells were determined by calculating the average CE values obtained per cycle, while those of the full‐cells were determined using the equation CE = (capacity retention)^1/cycle number^.

### Statistical Analysis

Average CEs were calculated as the mean of CE values from each cycle over 120 cycles. Overpotential, charge‐transfer resistance, and SEI resistance values presented in Table 1 are reported as mean ± SD from ten repeated measurements (*n* = 5) per condition. In Table 2, the average Li island diameter and inter‐island spacing were determined from 100 individual particles (*n* = 100) and 100 random inter‐island distances (*n* = 100), respectively, based on SEM images. Surface diffusivities were calculated from these measured geometric parameters. No data transformation, normalization, or outlier exclusion was applied. Data are presented as mean ± SD unless otherwise stated. Statistical analysis and figure generation were performed using OriginPro 2023.

## Conflict of Interest

The authors declare no conflict of interest.

## Supporting information



Supporting Information

## Data Availability

The data that support the findings of this study are available from the corresponding author upon reasonable request.

## References

[1] J. Qian , B. D. Adams , J. Zheng , W. Xu , W. A. Henderson , J. Wang , M. E. Bowden , S. Xu , J. Hu , J. G. Zhang , Adv. Funct. Mater. 2016, 26, 7094.

[2] D. Lin , Y. Liu , Y. Cui , Nat. Nanotechnol. 2017, 12, 194. 28265117 10.1038/nnano.2017.16

[3] C. Heubner , S. Maletti , H. Auer , J. Hüttl , K. Voigt , O. Lohrberg , K. Nikolowski , M. Partsch , A. Michaelis , Adv. Funct. Mater. 2021, 31, 2106608.

[4] X. Xu , Y. Liu , J.‐Y. Hwang , O. O. Kapitanova , Z. Song , Y.‐K. Sun , A. Matic , S. Xiong , Adv. Energy Mater. 2020, 10, 2002390.

[5] D. Aurbach , E. Zinigrad , Y. Cohen , H. Teller , Solid State Ionics 2002, 148, 405.

[6] J. G. Kim , D. Gu , K. H. Cho , C. Y. Im , S. J. Kim , Small 2023, 19, 2301207. 10.1002/smll.202301207 37154207

[7] D. Patrun , S. Zhao , Z. Aytuna , T. Fischer , M. Miess , Z. Hong , S. Mathur , Nano Energy 2024, 128, 109836.

[8] S. Jin , Y. Ye , Y. Niu , Y. Xu , H. Jin , J. Wang , Z. Sun , A. Cao , X. Wu , Y. Luo , H. Ji , L. J. Wan , J. Am. Chem. Soc. 2020, 142, 8818. 32310653 10.1021/jacs.0c01811

[9] X. Zeng , M. Mahato , W. Oh , H. Yoo , V. H. Nguyen , S. Oh , G. Valurouthu , S.‐K. Jeong , C. W. Ahn , Y. Gogotsi , I.‐K. Oh , Energy Environ. Mater. 2024, 7, 12686.

[10] J. Lee , Y.‐G. Cho , D. Gu , S. J. Kim , ACS Appl. Mater. Interfaces 2022, 14, 15080. 35227059 10.1021/acsami.1c21183

[11] E. Cha , M. D. Patel , J. Park , J. Hwang , V. Prasad , K. Cho , W. Choi , Nat. Nanotechnol. 2018, 13, 337. 29434261 10.1038/s41565-018-0061-y

[12] M. S. Whittingham , Prog. Solid State Chem. 1978, 12, 41.

[13] T. Stephenson , Z. Li , B. Olsen , D. Mitlin , Energy Environ. Sci. 2014, 7, 209.

[14] S. T. Oyakhire , W. Zhang , A. Shin , R. Xu , D. T. Boyle , Z. Yu , Y. Ye , Y. Yang , J. A. Raiford , W. Huang , J. R. Schneider , Y. Cui , S. F. Bent , Nat. Commun. 2022, 13, 3986. 35821247 10.1038/s41467-022-31507-w PMC9276694

[15] R. Xiong , Y. Yu , S. Chen , M. Li , L. Li , M. Zhou , W. Zhang , B. Yan , D. Li , H. Yang , Y. Zhang , H. Zhou , J. Power Sources 2023, 553, 232296.

[16] K. J. Vetter , in Electrochemical Kinetics: Theoretical Aspects (Ed.: K. J. Vetter ), Academic Press, San Diego, CA USA, 1967, pp. 104–395.

[17] A. Pei , G. Zheng , F. Shi , Y. Li , Y. Cui , Nano Lett. 2017, 17, 1132. 28072543 10.1021/acs.nanolett.6b04755

[18] A. Lodding , J. N. Mundy , A. Ott , Phys. Status Solidi 1970, 38, 559.

[19] M. Siniscalchi , J. Liu , J. S. Gibson , S. J. Turrell , J. Aspinall , R. S. Weatherup , M. Pasta , S. C. Speller , C. R. M. Grovenor , ACS Energy Lett. 2022, 7, 3593. 36277136 10.1021/acsenergylett.2c01793 PMC9578048

[20] E. Ganz , K. Sattler , J. Clarke , Surf. Sci. 1989, 219, 33.

[21] H. Martín , P. Carro , A. Hernández Creus , S. González , G. Andreasen , R. C. Salvarezza , A. J. Arvia , Langmuir 2000, 16, 2915.

[22] K. Roy , S. Maitra , D. Ghosh , P. Kumar , P. Devi , Chem. Eng. J. 2022, 435, 134963.

[23] C. J. Price , E. A. D. Baker , S. P. Hepplestone , J. Phys. Chem. C 2024, 128, 1867. 10.1021/acs.jpcc.3c05155 PMC10860140 38352854

[24] A. Jain , S. P. Ong , G. Hautier , W. Chen , W. D. Richards , S. Dacek , S. Cholia , D. Gunter , D. Skinner , G. Ceder , K. A. Persson , APL Mater. 2013, 1, 11002.

[25] A. Jain , G. Hautier , S. P. Ong , C. J. Moore , C. C. Fischer , K. A. Persson , G. Ceder , Matter Mater. Phys. 2011, 84, 45115.

[26] T. B. Massalski , J. L. Murray , Binary Alloy Phase Diagrams, American Society for Metals, Metals Park, OH 1986.

[27] D. R. Ely , R. E. García , J. Electrochem. Soc. 2013, 160, A662.

[28] Y. Wang , Y. Liu , M. Nguyen , J. Cho , N. Katyal , B. S. Vishnugopi , H. Hao , R. Fang , N. Wu , P. Liu , P. P. Mukherjee , J. Nanda , G. Henkelman , J. Watt , D. Mitlin , Adv. Mater. 2023, 35, 2206762. 10.1002/adma.202206762 36445936

[29] K. Qin , J. V. Nguyen , Z. Yang , C. Luo , Mater. Today Energy 2023, 31, 101199.

[30] Y. Hu , Z. Li , Z. Wang , X. Wang , W. Chen , J. Wang , W. Zhong , R. Ma , Adv. Sci. 2023, 10, 2206995. 10.1002/advs.202206995 PMC10131806 36806693

[31] T. T. K. Ingber , M. M. Bela , F. Püttmann , J. F. Dohmann , P. Bieker , M. Börner , M. Winter , M. C. Stan , J. Mater. Chem. A. 2023, 11, 17828.

[32] J. H. Lee , Y. G. Cho , D. Gu , S. J. Kim , ACS Appl. Mater. Interfaces 2022, 14, 15080. 35227059 10.1021/acsami.1c21183

[33] S. H. Lee , S. Y. Kim , S. M. Kim , J. I. Jeong , S. J. Kim , J. Alloys Compd. 2017, 704, 607.

