# Supplementary material for: Controlling Lithium Surface Diffusivity via 2D PtTe2, PdTe2, and NiTe2 Coatings for Anode‐Free and Lithium Metal Batteries
Source: Adv Mater. 2025 Jun 1;37(33):2501261. doi: 10.1002/adma.202501261 (PMC12369682; doi:10.1002/adma.202501261)
Supplement: Supplementary file 1 — Supporting Information [file ADMA-37-2501261-s001.pdf]

ADVANCED MATERIALS

Supporting Information

for *Adv. Mater.*, DOI 10.1002/adma.202501261

Controlling Lithium Surface Diffusivity via 2D $PtTe_2$, $PdTe_2$, and $NiTe_2$ Coatings for Anode-Free and Lithium Metal Batteries

*Chae Yoon Im, Ga Yeon Lee, Jong Gyeom Kim, Jeong Ho Choi and Suk Jun Kim**

Supporting Information

Controlling Lithium Surface Diffusivity via 2D $PtTe_2$, $PdTe_2$, and $NiTe_2$ Coatings for Anode-Free and Lithium Metal Batteries

*Chae Yoon Im, Ga Yeon Lee, Jong Gyeom Kim, Jeong Ho Choi, Suk Jun Kim**

School of Energy, Materials and Chemical Engineering, Korea University of Technology and Education, Cheonan, 31253 South Korea

E-mail: skim@koreatech.ac.kr

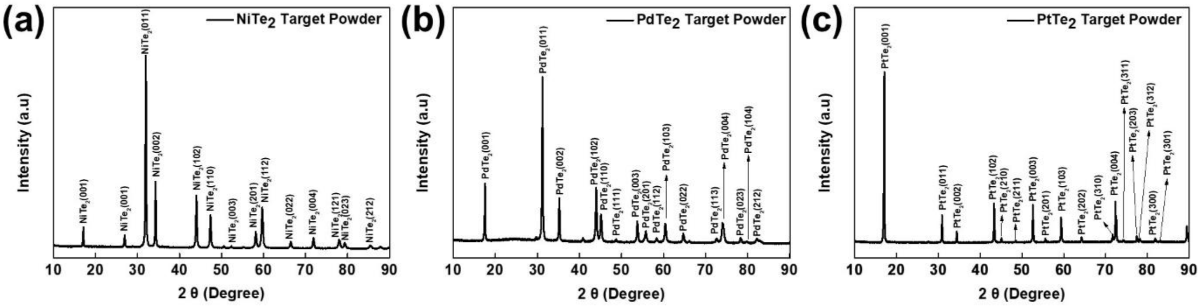

Figure S1. XRD profiles of $NiTe_2$, $PdTe_2$, and $PtTe_2$ powders used for fabrication of the sputtering targets.

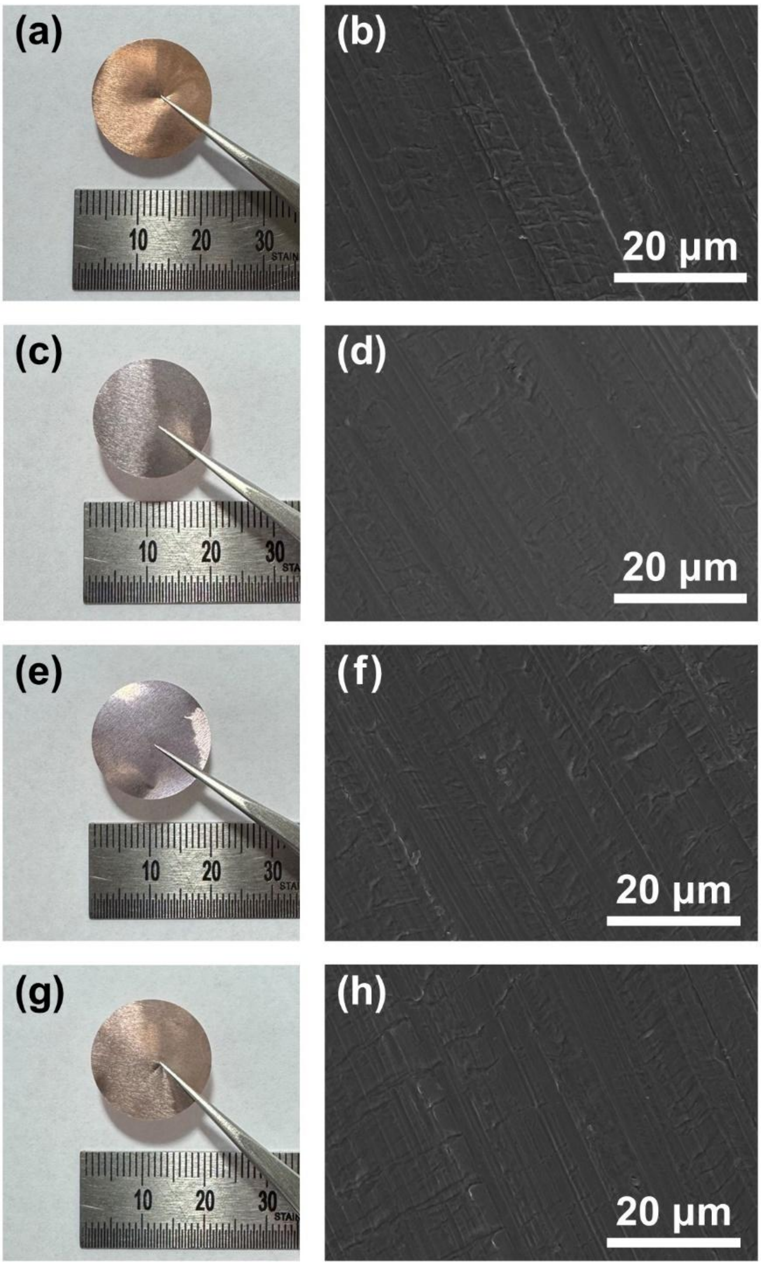

Figure S2. Digital images and SEM images of (a,b) Bare Cu, and bare Cu coated with (c,d) 10PtRT, (e,f) 15Pdh5, and (g,h) 10Nih10.

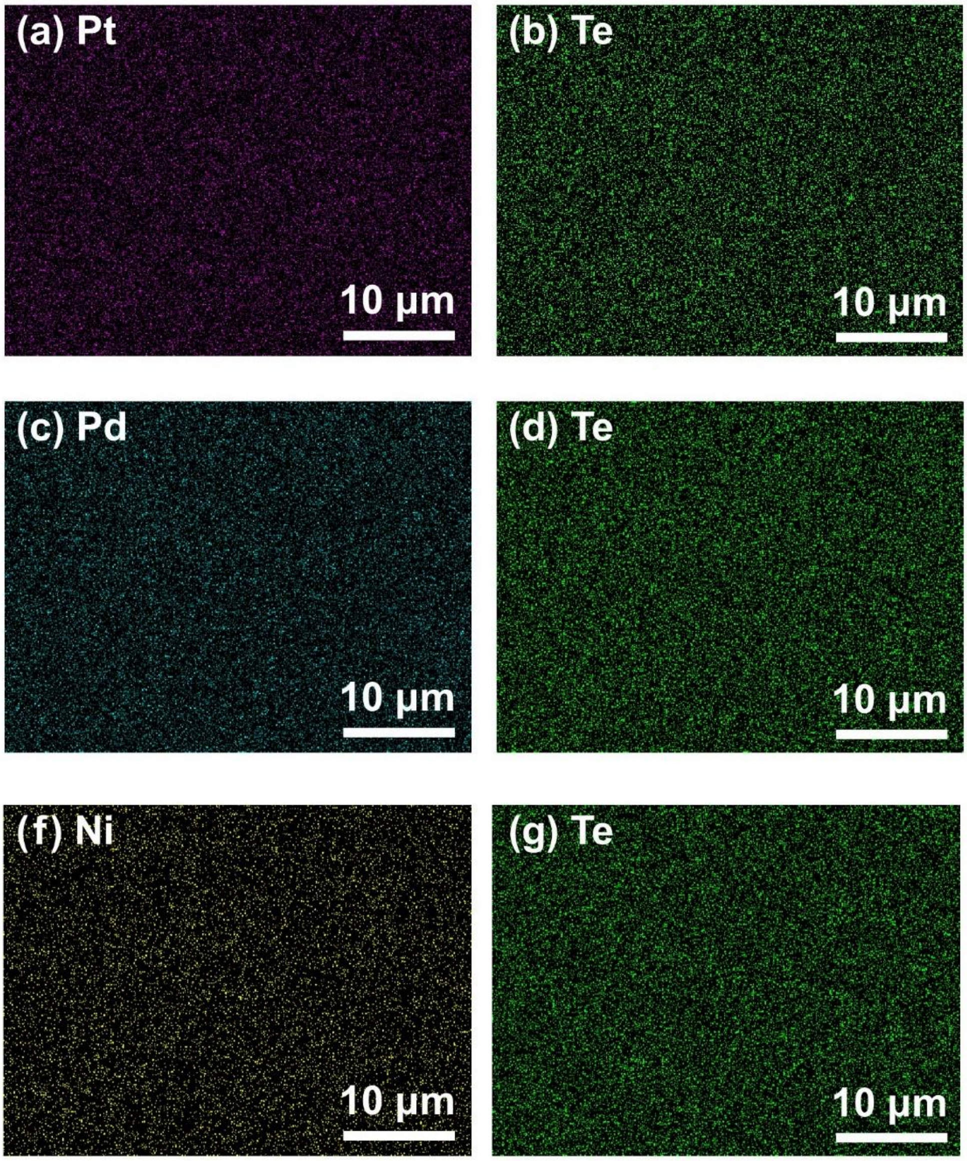

Figure S3. EDS mapping of the deposited thin films on Cu foils: (a) Platinum (Pt) and (b) Tellurium (Te) mapping for 10PtRT, (c) Palladium (Pd) and (d) Tellurium (Te) mapping for 15Pdh5, (f) Nickel (Ni) and (g) Tellurium (Te) mapping for 10Nih10.

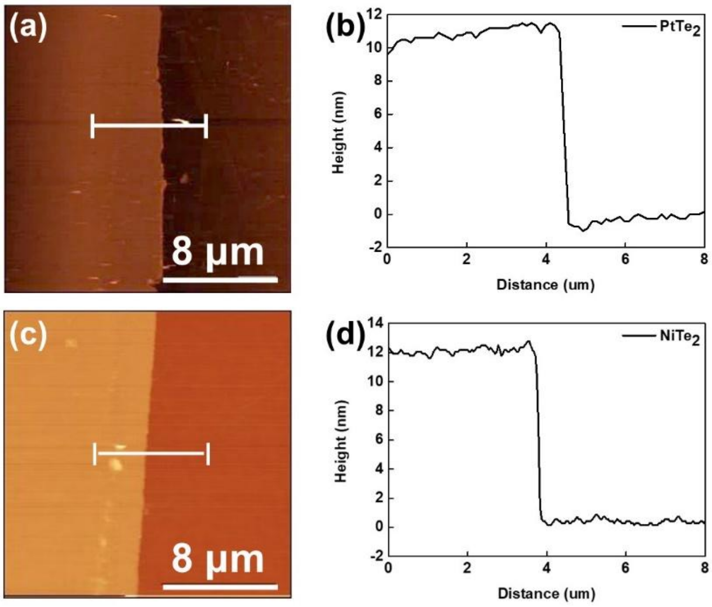

Figure S4. Measurement of $PtTe_2$ and $NiTe_2$ thin film thickness using AFM: (a, c) AFM topography images of $PtTe_2$ and $NiTe_2$ deposited on Si wafers, respectively. (b, d) Morphological profiles along the white lines indicated in the corresponding AFM images, showing the thickness of the thin films at their edges.

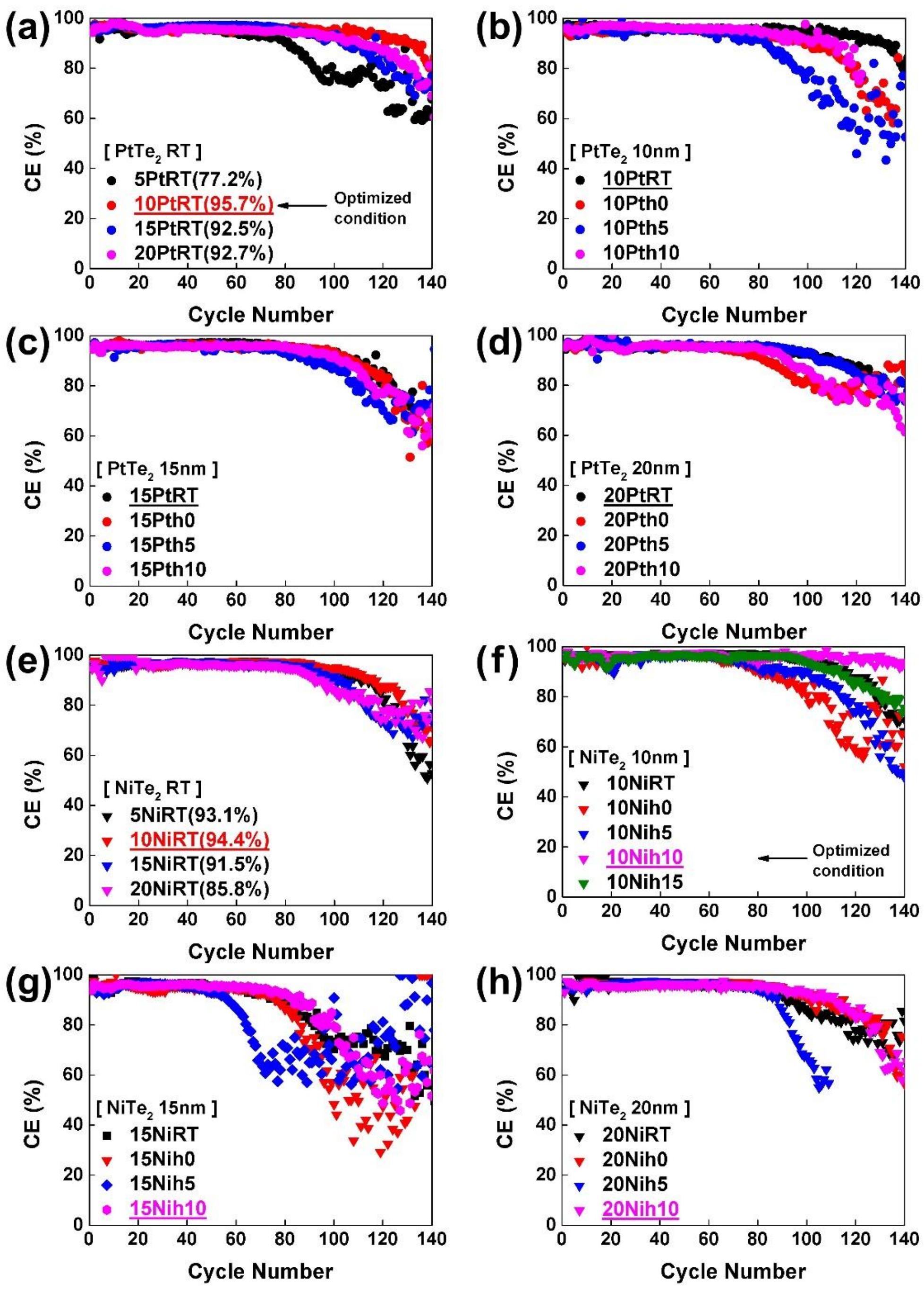

Figure S5. Optimizing coating conditions for half-cells through Coulombic Efficiency (CE) measurement: (a) CE as a function of $PtTe_2$ thickness at room temperature, and heat treatment time for (b) 10 nm $PtTe_2$, (c) 15 nm $PtTe_2$, and (d) 20 nm $PtTe_2$. Similarly, (e) CE as a function of $NiTe_2$ thickness at room temperature, and heat treatment time for (f) 10 nm $NiTe_2$, (g) 15 nm $NiTe_2$, and (h) 20 nm $NiTe_2$. CE values at the 100th cycle for $PtTe_2$ and $NiTe_2$ films without heating are indicated in the legends of (a) and (e), respectively. CE data for $PdTe_2$ were reported in our previous study.[1]

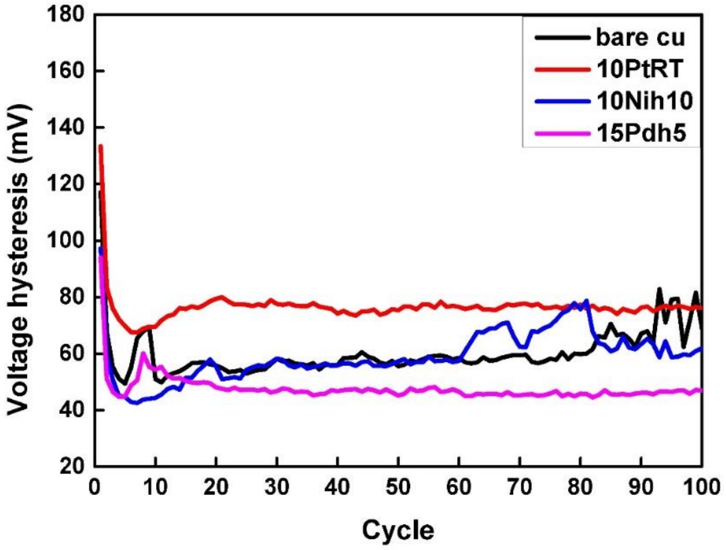

Figure S6. Voltage hysteresis of half-cells with bare Cu, 10PtRT-, 10Nih10-, and 15Pdh5-coated current collectors, corresponding to representative samples selected from the optimization results in Figure S5.

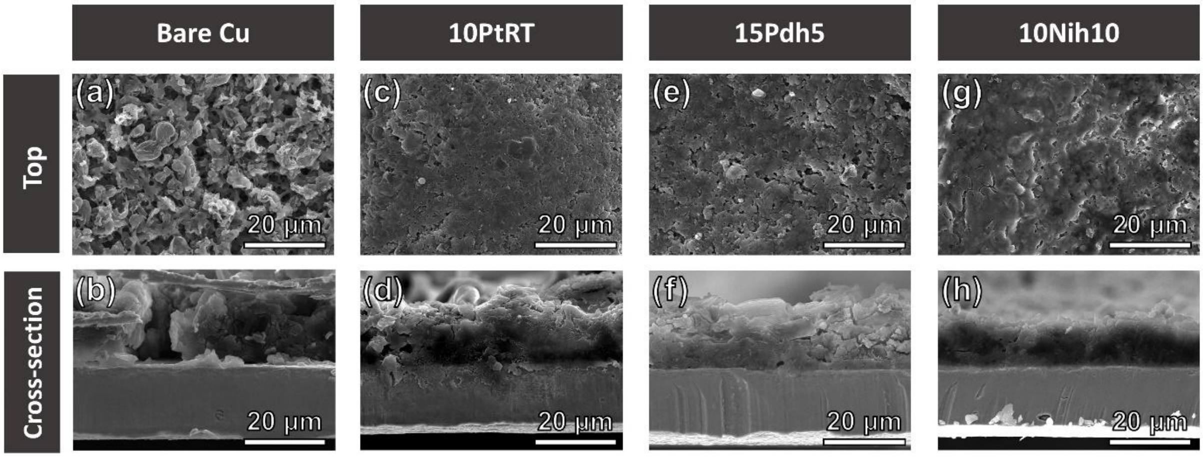

Figure S7. SEM images of Li deposition after the 150th plating (1 mAh cm^{-2}, 1 mA cm^{-2}): (a, c, e, g) Top-view and (b, d, f, h) cross-sectional SEM images of (a, b) bare Cu current collector (CC), (c, d) 10PtRT-, (e, f) 15Pdh5-, and (g, h) 10Nih10-coated CC.

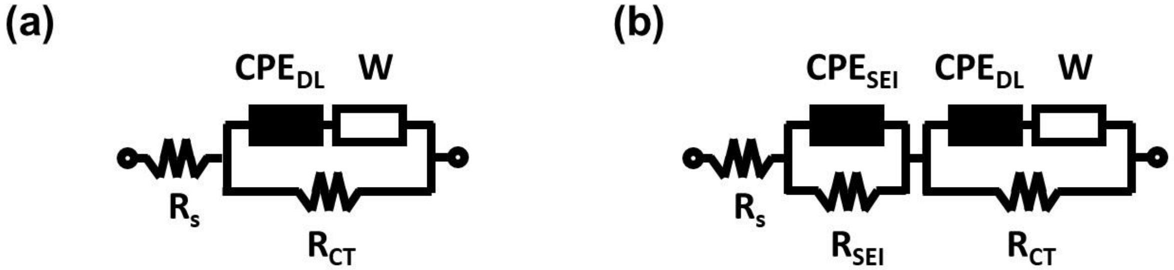

Figure S8. (a, b) The equivalent circuit diagrams for the EIS curves in Figure 3b and c, respectively.

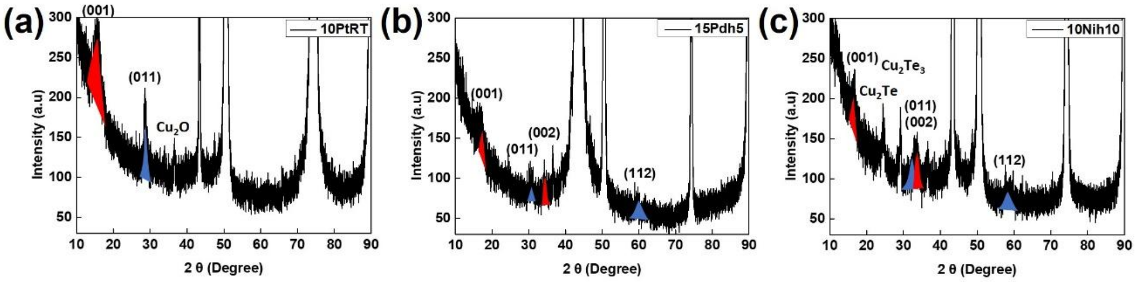

Figure S9. Indication of the XRD peak areas used for calculating the coverage area of grains with the (001) plane parallel to the substrate (001-grain). The coverage area ratio of 001-grain on the thin film is determined by the XRD peak area ratio, {(001) + (002)}/total peaks. The calculated peak area ratio for 10PtRT, 15Pdh5 and 10Nih10 are 75.1%, 63.4%, and 57.3%, respectively.

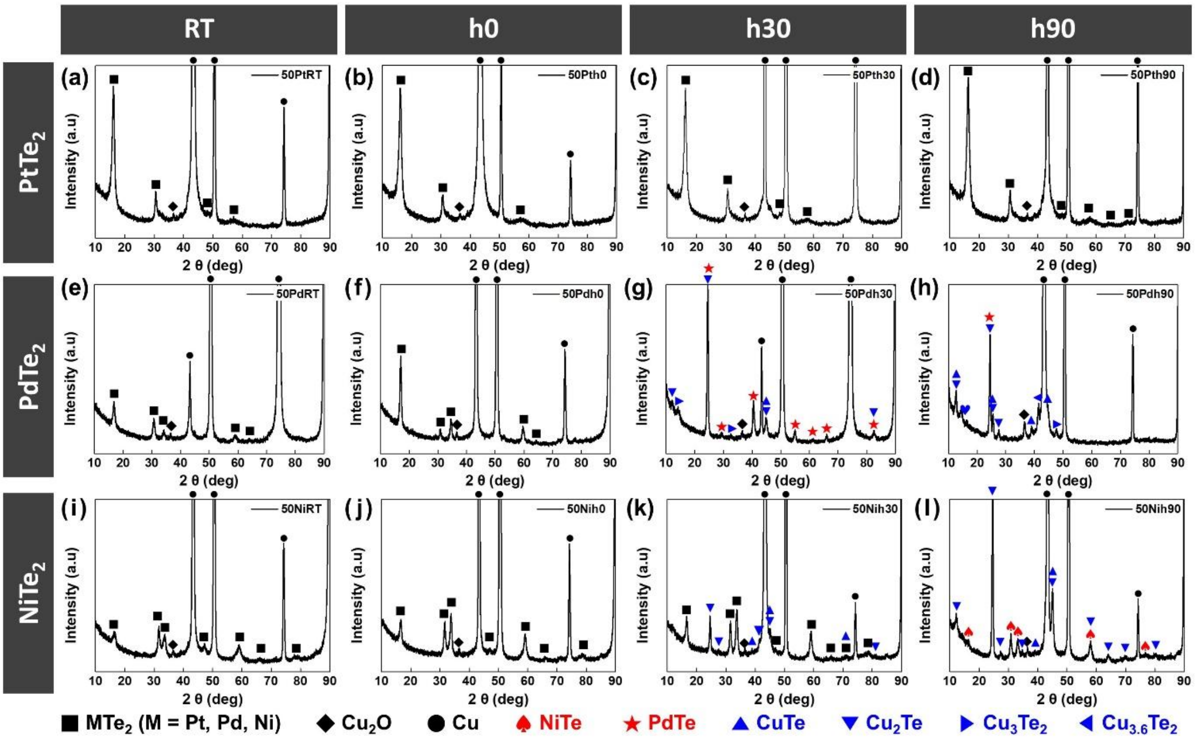

Figure S10. XRD profiles of (a) 50PtRT, (b) 50Pth0, (c) 50Nih30, (d) 50Nih90, (e) 50PdRT, (f) 50Pdh0, (g) 50Pdh30, (h) 50Pdh90, (i) 50NiRT, (j) 50Nih0, (k) 50Nih30, and (l) 50Nih90.

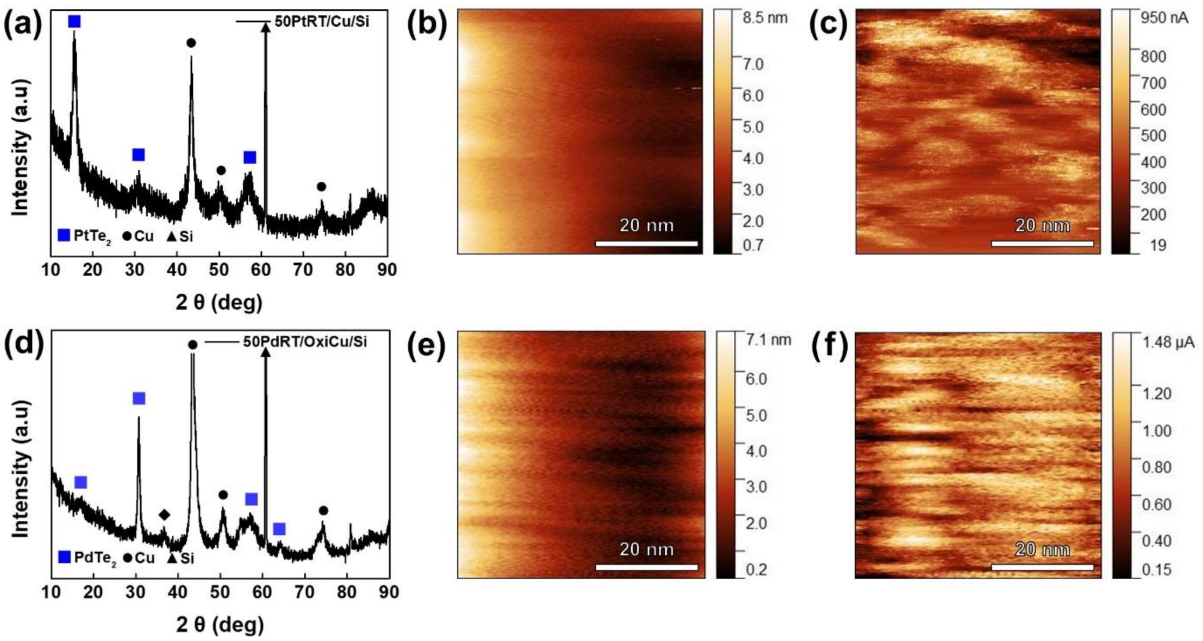

Figure S11. XRD profile of 50 nm-thick (a) $PtTe_2$ and (d) $PdTe_2$ deposited on Cu thin film deposited on Si wafer. (b,e) Topography and corresponding (c,f) current images from the (b,c) $PtTe_2$ and (e,f) $PdTe_2$ thin film measured in atomic force microscopy (AFM). The current is measured using the scanning spreading resistance microscopy (SSRM) mode of AFM. For the analysis, $PtTe_2$ and $PdTe_2$ were intentionally deposited on Si wafer to remove the effect of surface morphology of Cu foil. The ratio of dark area presenting high resistance area (that is 001-grain) on (c) $PtTe_2$ is about 56% and that is comparable to XRD peak ratio of 001-grain to others, 46.0±5.5%. Compatibility of area ratios of 001-grain measured in AFM and XRD is confirmed with PdTe2 thin-films (AFM:18% XRD: 17.4±3.5%). The coverage of 001-grain on 10PtRT deposited on a Cu thin film on a Si wafer differs from that on Cu foil (Figure S8 and S9). This suggests that the crystallographic orientation of 10PtRT is influenced by the underlying Cu layer, and differences in the fabrication processes between the Cu foil and Cu thin film may lead to variations in their crystallographic orientations.

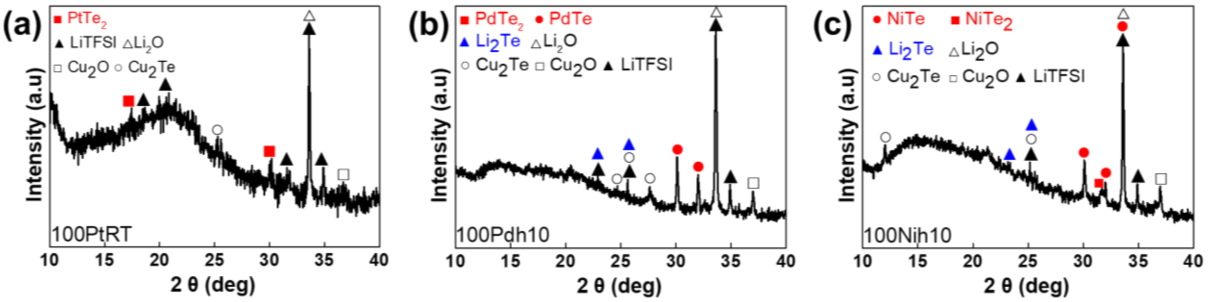

Figure S12. XRD analyses of the CC coated with 100 nm-thick (a) 100PtRT, (b) 100Pdh5, and (c) 100Nih10 thin-films after 50 cycles in the half-cell configuration (1.0 mAh cm^{-2} @ 0.1 mA cm^{-2}).

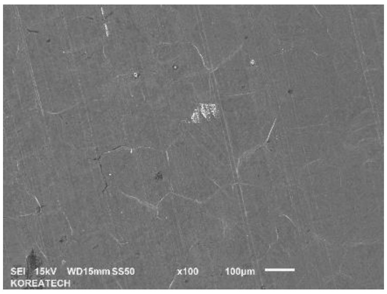

Figure S13. SEM image of the top surface of as-received bare Li without coating or immersion in the electrolyte.

Tables

Table S1. Comparison of XRD peak area ratio of 50 nm-thick $PtTe_2$, $PdTe_2$, and $NiTe_2$ thin films prepared under three different conditions: without heating (RT), heating during sputtering only (h0), and during sputtering followed by an additional 30 min heating (h30). These ratios are further compared to the peak intensity ratios from powder diffraction data obtained from the ICSD.

50nm XRD		Area ratio (%) (ICSD : Intensity ratio)			
		(001+002)/all	(011)/all	(110)/all	(112)/all
$PtTe_2$	ICSD	21.4	78.6	-	-
	RT	78.5 ± 1.04	21.5 ± 1.04	-	-
	h0	84.0 ± 0.61	16.0 ± 0.61	-	-
	h30	76.5 ± 0.57	23.5 ± 0.57	-	-
$PdTe_2$	ICSD	20.2	78.9	-	4.5
	RT	55.9 ± 2.14	31.7 ± 2.71	-	12.3 ± 0.59
	h0	76.8 ± 0.80	6.7 ± 0.82	-	16.5 ± 0.54
$NiTe_2$	ICSD	13.6	57.7	22.3	8.9
	RT	40.5 ± 0.93	32.1 ± 1.79	5.1 ± 0.93	22.3 ± 0.70
	h0	48.6 ± 1.45	24.5 ± 1.30	2.2 ± 0.52	24.7 ± 1.94
	h30	49.5 ± 0.82	21.1 ± 0.63	2.2 ± 0.53	27.1 ± 0.72

For further understanding the phase transition, grain growth, and crystallographic orientation with respect to substrate, the thicker 2D thin films are analyzed to achieve XRD signal with higher intensity after longer annealing time as shown in Figure S9. 50 nm thick $PtTe_2$, $PdTe_2$, and $NiTe_2$ thin films deposited on Cu CC were prepared without heating (RT), with in situ heating (h0), and with in situ heating followed by additional heating for 30 min (h30) at 473 K. 50 nm-thick $PtTe_2$ thin film that is in situ annealed followed by additional annealing for 90 min (50Pth90) maintained its structural integrity, without undergoing any phase transition, forming no Cu-Te phases, and showing no significant grain growth. This observation underscores the material's thermal stability and resistance to structural

changes under prolonged heat treatment. In contrast, $PdTe_2$ entirely transformed into PdTe forming Cu-Te phases when heating duration is longer than 30 min (50Pdh30) though $PdTe_2$ phase was maintained during sputtering with in situ heating (50Pdh0). In case of $NiTe_2$, $NiTe_2$ phase was maintained up to 30 min heating (50Nih30), yet totally transformed into NiTe forming Cu-Te phases after 90 min annealing (50Nih90).

Table S2. Comparison of electrochemical performance of anode-free Li ion batteries.

Material	Thickness	Electrolyte	Cathode active materials (Capacity)	Capacity Retention (1st discharge capacity)	Ref
**$PtTe_2$/Cu**	10 nm	1 M LiTFSI in DOL/DME (1:1 v/v) with 2 wt % $LiNO_3$	LFP (1 mAh/cm^2)	75.3 % after 100 cycles at 0.2 C (99.9 mAh/g)	This Work
**LSM-Cu**	10 – 15 nm	1 M $LiPF_6$ in EC/EMC (3:7 v/v) with 2 wt % VC	LFP (3 mAh/cm^2, pouch cell)	83.7 % after 166 cycles at 0.02 C (200 mAh/g)	[2]
**CTN-60/Cu**	Not found	1 M LiTFSI in DOL/DME (1:1 v/v) with 2 wt % Li_2S 2 wt % $LiNO_3$	NFP (2 mAh/cm^2)	85.98 % after 100 cycles at 2 C (Not-found)	[3]
**3DPI@Au**	10 um	1 M $LiPF_6$ in EC/DME (1:1 v/v) with 10 wt % FEC and 1 wt % VC	NCM811 (6.4 mAh/cm^2)	86.0 % after 100 cycles at 0.2C (Not-found)	[4]
**C-SEI/Cu**	Not-found	1 M LiTFSI in DOL/DME (1:1 v/v) with 2 wt % $LiNO_3$	LFP (14 mg/cm^2)	62.3 % after 100 cycles at 0.2C (-)	[5]
**CPD/Cu**	16 – 17 nm	1 M LiTFSI in DOL/DME (1:1 v/v) with 0.2 M $LiNO_3$	$LiFePO_4$ (Not-found)	63 % after 100 cycles at 0.3C (147 mAh/g)	[1]
**Ag@Zr-DMBC/Cu**	18 um	1 M LiTFSI in DOL/DME (1:1 v/v) with 1 % $LiNO_3$	LFP (4.3 mg/cm^2)	99.3 % after 1000 cycles at 1C (159.8 mAh/g)	[6]
**12-MG/Cu**	12 nm	1 M LiTFSI in DOL/DME (1:1 v/v) with 2 wt % $LiNO_3$	LFP (1 mAh/cm)	63.6 % after 100 cycles at 0.2C (125.9 mAh/g)	[7]
**Alloy-type /Cu**	20 nm	2 M LiDFOB EC/DEC (1:1 v/v)	NCM622 (3 mAh/cm^2)	46.8 % after 30 cycles at Not-found (Not-found)	[8]
**Cu@FSL**	Not-found	1 M $LiPF_6$ in EC/DMC (1:1 v/v) with 5 vol% FEC	NCM811 (10 mg/cm^2)	60.9 % after 100 cycles at 0.2 mA/cm^2 (190 mAh/g)	[9]

Table S3 Comparison of electrochemical performance of Lithium Metal batteries.

**Material**	**Thickness**	**Electrolyte**	**Cathode active materials (Capacity)**	**Capacity Retention (1st discharge capacity)**	**Ref.**
**$5PdTe_2$**	5 nm	1 M LiTFSI in DOL/DME (1:1 v/v) with 2 wt % $LiNO_3$	LFP (1.5 mAh/cm^2) LFP (2.0 mAh/cm^2) LFP (2.5 mAh/cm^2) LFP (3.0 mAh/cm^2)	87.5 % after 200 cycles at 0.5 C (160 mAh/g) 87.5 % after 200 cycles at 0.5 C (160 mAh/g) 87.5 % after 200 cycles at 0.5 C (160 mAh/g) 87.5 % after 200 cycles at 0.5 C (160 mAh/g)	This work
**BF_3-doped monolayer Ti_3C_2**	Not-found	1 M LiTFSI in DOL/DME(1:1 v/v) with 1 wt % $LiNO_3$	NCA (-)	83.7% after 200 cycles at 0.1 C, previously 4 cycles at 0.2 C (182.3 mAh/g)	[10]
**TPU**	5 um	1 M LiTFSI in DOL/DME (1:1 v/v) with 2 wt% $LiNO_3$	LFP (Not-found)	87.5 % after 200 cycles at 0.5 C (160 mAh/g)	[11]
**CS/DF-PEG-DF**	180 nm	1 M $LiPF_6$ in EC/DMC/EMC (1:1:1 v/v)	LFP (20 mg/cm^2)	60.1 % after 100 cycles at 0.5 C, previously 5 cycles at 0.1 C (Not-found)	[12]
**Li-B(composite)@ssm**	Total 47 um	1 M $LiPF_6$ in EC/DMC/EMC (3:7:1 v/v)	LFP (14 mg/cm^2)	97.5 % after 400 cycles at 1 C (147 mAh/g)	[13]
**Li@L/LN**	8 um	0.5 M LiTFSI in DOL/DME (1:1 v/v) with 0.5 M HNO_3	Sulfur/Carbon (1 mg/cm^2)	Not-found after 300 cycles at 0.2 C, previously 2 cycles at 0.1 C (Not-found)	[14]
**PZEM**	15 um	1 M $LiPF_6$ in EC/DEC (1:1 v/v)	LFP (4.2 mg/cm^2) NMC811 (8.4 mg/cm^2)	- LFP 99.6 % after 400 cycles at 0.5 C (151.9 mAh/g) 83 % after 1000 cycles at 2C (136.3g) - NCM811 94.3fter 150 cycles at 0.2C, previously 2 cycles at 0.5 C	[15]

				(178.4 mAh/g)	
**LiBr@Li**	2 um	1 M LiTFSI in DOL/DME (1:1 v/v) with 0.2 M $LiNO_3$	LFP (4.0 mg/cm^2)	88.1 % after 500 cycles at 0.5 C (Not-found)	[16]
**COF-COOH@PP separator**	10 um	1 M LiTFSI in DOL/DME (1:1 v/v) with 5 wt% $LiNO_3$	LFP (Not-found)	Not-Found (170 mAh/g)	[17]
**Zr-LMA**	12 nm	1 M LiTFSI in DOL/DME (1:1 v/v) with 2 wt % $LiNO_3$	LFP (1.5 mAh/cm^2)	66.6 % after 1500 cycles at 1 C (137.5 mAh/g)	[7]

References

[1] J. H. Lee, Y. G. Cho, D. Gu, S. J. Kim, 2D PdTe2Thin-Film-Coated Current Collectors for Long-Cycling Anode-Free Rechargeable Batteries, *ACS Appl. Mater. Interfaces* **2022**, *14*, 15080.

[2] D. Aurbach, A. Zaban, Y. Ein-Eli, I. Weissman, O. Chusid, B. Markovsky, M. Levi, E. Levi, A. Schechter, E. Granot, Recent Studies on the Correlation between Surface Chemistry, Morphology, Three-Dimensional Structures and Performance of Li and Li-C Intercalation Anodes in Several Important Electrolyte Systems, *J. Power Sources* **1997**, *68*, 91.

[3] A. Manthiram, Y. Fu, S. Chung, C. Zu, Y. Su, Rechargeable Lithium − Sulfur Batteries, **2014**.

[4] K. Xu, Nonaqueous Liquid Electrolytes for Lithium-Based Rechargeable Batteries, *Chem. Rev.* **2004**, *104*, 4303.

[5] P. Biswal, S. Stalin, A. Kludze, S. Choudhury, L. A. Archer, Nucleation and Early Stage Growth of Li Electrodeposits, *Nano Lett.* **2019**, *19*, 8191.

[6] J. G. Kim, D. Gu, K. H. Cho, C. Y. Im, S. J. Kim, Exploiting Zirconium-Based Metallic Glass Thin Films for Anode-Free Lithium-Ion Batteries and Lithium Metal Batteries With Ultra-Long Cycling Life, *Small* **2023**, *19*, 1.

[7] T. T. K. Ingber, M. M. Bela, F. Püttmann, J. F. Dohmann, P. Bieker, M. Börner, M. Winter, M. C. Stan, Elucidating the Lithium Deposition Behavior in Open-Porous Copper Micro-Foam Negative Electrodes for Zero-Excess Lithium Metal Batteries, *J. Mater. Chem. A* **2023**, *11*, 17828.

[8] A. A. Assegie, J. H. Cheng, L. M. Kuo, W. N. Su, B. J. Hwang, Polyethylene Oxide Film Coating Enhances Lithium Cycling Efficiency of an Anode-Free Lithium-Metal Battery, *Nanoscale* **2018**, *10*, 6125.

[9] T. T. Beyene, B. A. Jote, Z. T. Wondimkun, B. W. Olbassa, C. J. Huang, B. Thirumalraj, C. H. Wang, W. N. Su, H. Dai, B. J. Hwang, Effects of Concentrated Salt and Resting Protocol on Solid Electrolyte Interface Formation for Improved Cycle Stability of Anode-Free Lithium Metal Batteries, *ACS Appl. Mater. Interfaces* **2019**, *11*, 31962.

[10] Z. Zhang, H. Luo, Z. Liu, S. Wang, X. Zhou, Z. Liu, A Chemical Lithiation Induced Li4.4Sn Lithiophilic Layer for Anode-Free Lithium Metal Batteries, *J. Mater. Chem. A* **2022**, *10*, 9670.

[11] T. M. Hagos, T. T. Hagos, H. K. Bezabh, G. B. Berhe, L. H. Abrha, S. F. Chiu, C. J. Huang, W. N. Su, H. Dai, B. J. Hwang, Resolving the Phase Instability of a Fluorinated Ether, Carbonate-Based Electrolyte for the Safe Operation of an Anode-Free Lithium Metal Battery, *ACS Appl. Energy Mater.* **2020**, *3*, 10722.

[12] C. Shan, Z. Qin, Y. Xie, X. Meng, J. Chen, Y. Chang, R. Zang, L. Wan, Y. Huang, Cu-CNTs Current Collector Fabricated by Deformation-Driven Metallurgy for Anode-Free Li Metal Batteries, *Carbon N. Y.* **2023**, *204*, 367.

[13] K. Qin, J. V. Nguyen, Z. Yang, C. Luo, Anion Modification for Stable Solid Electrolyte Interphase in Anode-Free Lithium Metal Batteries, *Mater. Today Energy* **2023**, *31*, 101199.

[14] H. yi Xia, Y. ke Wang, Z. wen Fu, Growing Cuprite Nanoparticles on Copper Current Collector toward Uniform Li Deposition for Anode-Free Lithium Batteries, *Appl. Surf. Sci.* **2023**, *617*, 156529.

[15] Y. Hu, Z. Li, Z. Wang, X. Wang, W. Chen, J. Wang, W. Zhong, R. Ma, Suppressing Local Dendrite Hotspots via Current Density Redistribution Using a Superlithiophilic Membrane for Stable Lithium Metal Anode, *Adv. Sci.* **2023**, *10*, 1.

[16] X. Yao, J. Wang, S. Lin, C. Tao, X. Zhang, W. Wang, C. Zhao, L. Wang, J. L. Bao, Y. Wang, T. Liu, Surface Bromination of Lithium-Metal Anode for High Cyclic Efficiency, *Adv. Energy Mater.* **2023**, *13*, 1.

[17] Q. An, H. en Wang, G. Zhao, S. Wang, L. Xu, H. Wang, Y. Fu, H. Guo, Understanding Dual-Polar Group Functionalized COFs for Accelerating Li-Ion Transport and Dendrite-Free Deposition in Lithium Metal Anodes, *Energy Environ. Mater.* **2023**, *6*, 1.
